# TIPRL Regulates Stemness and Survival in Lung Cancer Stem Cells through CaMKK2‐CaMK4‐CREB Feedback Loop Activation

**DOI:** 10.1002/advs.202406309

**Published:** 2024-07-30

**Authors:** In‐Sung Song, Yu‐Jeong Jeong, Jae Kwang Yun, Jimin Lee, Hae‐Jun Yang, Young‐Ho Park, Sun‐Uk Kim, Seung‐Mo Hong, Peter C.W. Lee, Geun Dong Lee, Sung‐Wuk Jang

**Affiliations:** ^1^ Department of Biochemistry and Molecular Biology Brain Korea 21 Project Asan Medical Center University of Ulsan College of Medicine Seoul 138‐736 Republic of Korea; ^2^ Department of Thoracic and Cardiovascular Surgery Asan Medical Center University of Ulsan College of Medicine Seoul 138‐736 Republic of Korea; ^3^ Futuristic Animal Resource & Research Center Korea Research Institute of Bioscience and Biotechnology Chungchenongbuk‐do 28116 Republic of Korea; ^4^ Department of Functional Genomics KRIBB School of Bioscience Korea University of Science and Technology (UST) Daejeon 34113 Republic of Korea; ^5^ Department of Pathology Asan Medical Center University of Ulsan College of Medicine Seoul 138‐736 Republic of Korea

**Keywords:** CaMKK2‐CaMK4‐CREB feedback loop activation, cancer stem cell, drug resistance, lung cancer, metastasis, TIPRL

## Abstract

Frequent recurrence and metastasis caused by cancer stem cells (CSCs) are major challenges in lung cancer treatment. Therefore, identifying and characterizing specific CSC targets are crucial for the success of prospective targeted therapies. In this study, it is found that upregulated TOR Signaling Pathway Regulator‐Like (TIPRL) in lung CSCs causes sustained activation of the calcium/calmodulin‐dependent protein kinase kinase 2 (CaMKK2) signaling pathway by binding to CaMKK2, thereby maintaining stemness and survival. CaMKK2‐mediated activation of CaM kinase 4 (CaMK4) leads to phosphorylation of cAMP response element‐binding protein (CREB) at Ser129 and Ser133, which is necessary for its maximum activation and the downstream constitutive expression of its target genes (*Bcl2* and *HMG20A*). TIPRL depletion sensitizes lung CSCs to afatinib‐induced cell death and reduces distal metastasis of lung cancer in vivo. It is determined that CREB activates the transcription of TIPRL in lung CSCs. The positive feedback loop consisting of CREB and TIPRL induces the sustained activation of the CaMKK2‐CaMK4‐CREB axis as a driving force and upregulates the expression of stemness‐ and survival‐related genes, promoting tumorigenesis in patients with lung cancer. Thus, TIPRL and the CaMKK2 signaling axis may be promising targets for overcoming drug resistance and reducing metastasis in lung cancer.

## Graphical abstract

The CREB‐TIPRL positive feedback regulation loop helps maintain the stemness and survival of lung CSCs. TIPRL induces sustained activation of the CaMKK2‐CaMK4‐CREB axis. In turn, the activated CREB upregulates the expression of TIPRL, Bcl2, and HMG20A to maintain the stemness and survival of CSCs, thus promoting tumorigenesis in lung cancer.

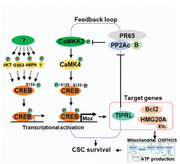

## 1 Introduction

Lung cancer is a major cause of mortality worldwide. In 2021, 235760 new cases of lung cancer were diagnosed in the United States, with over 100 000 mortalities.^[^ ^1^ ^]^ Fortunately, due to advancements in cancer biology and techniques, the incidence and mortality of lung cancer have declined over the past two decades.^[^ ^2^ ^]^ Specifically, the development and introduction of inhibitors of well‐known protein kinases, such as epidermal growth factor receptor (EGFR), mitogen‐activated protein kinase kinase 1/2, cyclin‐dependent kinase 4/6, and anaplastic lymphoma kinase, have considerably improved outcomes.^[^ ^3^ ^]^ These drugs have remarkable advantages over classical chemotherapeutic agents since they act on disease‐specific pathways related to signal transduction.^[^ ^4^ ^]^ Imatinib was the first protein kinase inhibitor to receive US Food and Drug Administration approval in 2001. Since then, 76 drugs have been approved for clinical use as of 2021, mainly for the treatment of various cancers.^[^ ^5^ ^]^ However, despite the development of tailored anticancer drugs and treatments, recurrence and metastasis of lung cancer are still frequent because of the properties of tumor cells. Moreover, lung cancer cells can acquire resistance to kinase inhibitors within ≈12–18 months through kinase mutations or alternative pathways.^[^ ^6^, ^7^ ^]^

Initially, tumorigenic ability, heterogeneity, and adaptive plasticity were considered general properties of solid tumors,^[^ ^8^, ^9^, ^10^ ^]^ but these are now considered characteristics of cancer stem cells (CSCs), which sets them apart from bulk tumor cells. CSCs have been implicated in cancer relapse, metastasis, multidrug resistance, and radiation resistance. The fate of CSCs can be altered by microenvironmental signals. As a result, anticancer drugs may activate quiescent CSCs into proliferating CSCs, initiating tumor growth and recurrence.^[^ ^11^ ^]^ Thus, therapeutic agents must target both quiescent and proliferating CSCs, as well as CSC populations arising from specific mutations and cells with acquired mutations that eliminate dependence on a single stem cell pathway.^[^ ^12^ ^]^

The activation of protein phosphatase 2A (PP2A) was recently reported to suppress small cell lung CSCs via protein kinase A inhibition,^[^ ^13^ ^]^ thus inhibiting the expansion of breast CSCs.^[^ ^14^ ^]^ The combined inhibition of a kinase and activation of a phosphatase, rather than targeting the kinase alone, can possibly yield more durable and potent inhibition. PP2A is a major tumor suppressor that plays an important role in cell cycle progression, DNA damage response, and apoptosis. In cancer cells, PP2A is rarely mutated and is predominantly inactivated by non‐genetic mechanisms.^[^ ^15^ ^]^ The non‐genetic and selective inhibition of PP2A by inhibitor proteins allows for therapeutic reactivation of the tumor suppressor activity of PP2A.^[^ ^16^, ^17^ ^]^

TOR signaling pathway regulator‐like (TIPRL) is a negative regulator of the catalytic subunit of PP2A (PP2Ac). It forms a complex together with a4, which is independent of the scaffold and regulatory subunits of PP2A.^[^ ^18^, ^19^, ^20^ ^]^ TIPRL appears to play a complex role in various cellular processes, including mammalian target of rapamycin signaling, DNA damage, autophagy, and cell death. Its interaction with PP2Ac and various signaling pathway‐related kinases suggests that it may be involved in the regulation of multiple signaling pathways in different types of cancers. The identification of novel TIPRL binding partners could provide valuable insights into the regulatory mechanisms underlying these cellular processes and further our understanding of the properties of lung CSCs.

This study aimed to identify and characterize specific CSC targets in order to design prospective targeted therapies that can overcome the frequent recurrence and metastasis of lung cancer due to CSCs. We identified a novel binding partner of TIPRL, calcium/calmodulin‐dependent protein kinase kinase 2 (CaMKK2). We found that TIPRL regulates the CaMKK2‐calcium/calmodulin‐dependent protein kinase 4 (CaMK4)‐cAMP response element‐binding protein (CREB) signaling axis by blocking the interaction between PP2A and CaMKK2 in lung CSCs. We believe that our findings can elucidate the regulatory mechanisms underlying the action of TIPRL in lung CSCs, as well as provide potential therapeutic targets for the treatment of lung cancer.

## 2 Results

### 2.1 TIPRL is Upregulated in Lung Cancer and Involved in CaMKK2 Signaling

**Figure 1:**
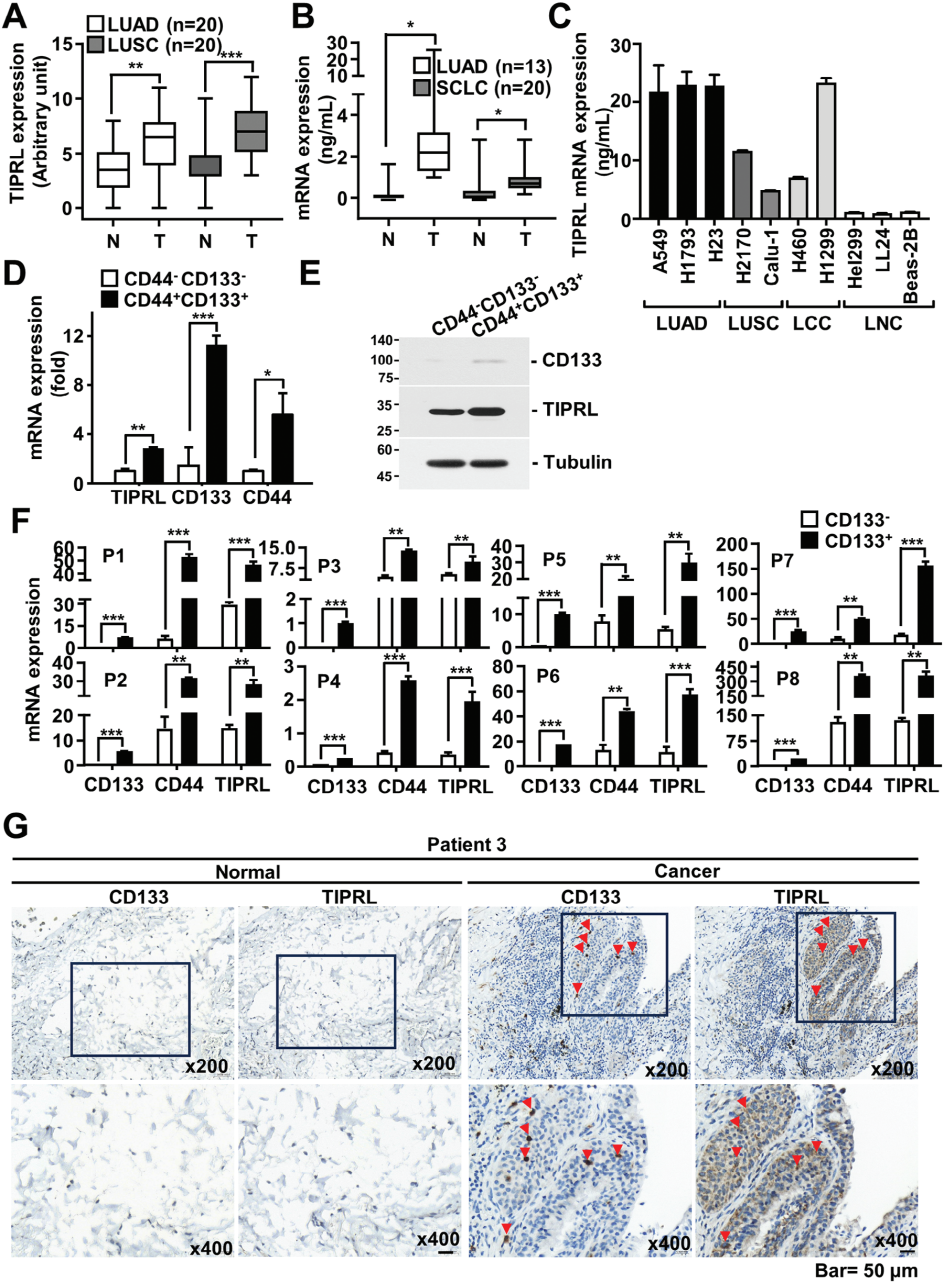
TIPRL expression is upregulated in lung cancer tissues and CSCs, correlating with poor prognosis. A) TIPRL levels in the tissues of patients with LUAD and LUSC compared with those in adjacent normal tissues, measured using immunoblotting (Figure S1A,B, Supporting Information); TIPRL band intensity was normalized to that of tubulin for each patient. B) TIPRL transcript levels in the tissues of patients with LUAD and SCLC, measured using qRT‐PCR (Figure S1C,D, Supporting Information); results were calculated as the ratio of the transcript level in the cancer tissue to that in the non‐cancer tissue after normalizing against the B2M level in each patient. C) TIPRL expression in lung cancer cell lines. TIPRL D) mRNA and E) protein levels in CD44^+^CD133^+^ and CD44^–^CD133^–^ A549 cells, analyzed using qRT‐PCR and immunoblotting, respectively. F) Transcription levels of TIPRL, CD133, and CD44 in freshly isolated CSCs from lung cancer tissues, analyzed using qRT‐PCR; the mRNA level of each gene was normalized to that of B2M in each sample. G) TIPRL and CD133 expression in lung cancer and adjacent normal tissues, assessed using immunohistochemistry; red arrows indicate highly positive cells. TIPRL, target of rapamycin signaling pathway regulator; CD, cluster of differentiation; LUAD, lung adenocarcinoma; SCLC, small cell lung cancer; LUSC, lung squamous cell carcinoma; CSCs, cancer stem cells; B2M, β2‐microglobulin; qRT‐PCR, quantitative reverse‐transcription PCR.

To determine whether TIPRL involved in stemness and anticancer drug resistance as a target for eliminating lung CSCs, we examined its expression in lung cancer tissues, including lung adenocarcinoma (LUAD), lung squamous cell carcinoma (LUSC), and small cell lung cancer (SCLC). Cancerous tissues displayed an increase in both the mRNA and protein levels of TIPRL (Figure S1A–1D, Supporting Information; **Figure** **1**A,B). TIPRL also displayed high expression in several lung cancer cell lines, as compared to that in normal epithelial cell lines (Figure 1C; Figure S1E, Supporting Information). Furthermore, we confirmed whether the level of TIPRL expression affects the survival rate of patients with lung cancer, as determined via cohort analysis of The Cancer Genome Atlas (TCGA). Patients with high TIPRL expression had a lower survival rate compared to those with low TIPRL expression (Figure S1F, Supporting Information).

TIPRL expression was examined in lung CSCs to investigate whether it is a potential regulator of CSC stemness and survival. TIPRL expression was significantly increased in CSCs isolated from lung cancer cell lines using cluster of differentiation (CD)133 and CD44 antibodies, compared to non‐CSCs (Figure 1D,E; Figure S2A, Supporting Information). Next, we compared the patterns of TIPRL expression between the cells isolated using an anti‐CD133 antibody and a combination of CD133 and CD44 antibodies, which yielded similar results (Figure 1D,E; Figure S2B, Supporting Information). Additionally, we demonstrated that tumorigenic ability was similar between the CD133^+^ population and CD44^+^CD133^+^ population cells (Figure S2C,D, Supporting Information). These findings indicate that CD133^+^ CSCs are identical to cells highly positive for CD44. Therefore, the cell populations isolated with CD133 antibody were used to conduct functional studies in the lung CSCs, such as western blotting, mitochondrial activity, quantitative reverse‐transcription PCR (qRT‐PCR), and immunocytochemistry. Fluorescence‐activated cell sorting analysis after co‐staining with CD133‐allophycocyanin and TIPRL‐fluorescein isothiocyanate (FITC) antibodies revealed elevated TIPRL expression in CD133^+^ cells (Figure S2E, Supporting Information). Additionally, qRT‐PCR demonstrated increased TIPRL expression in CD133^+^ CSCs freshly isolated from patients with lung cancer (Figure 1F). TIPRL and CD133 mRNA expression levels in cells from patient tissues were strongly correlated (Figure S2F, Supporting Information). Finally, immunohistochemistry was used to demonstrate that the TIPRL protein correlated with the strong signal observed in the adjacent tumor region stained with an anti‐CD133 antibody (Figure 1G; Figure S2G,H, Supporting Information). These findings suggest that TIPRL plays a critical role in lung CSCs and lung carcinogenesis.

We then investigated which kinases are regulated upon TIPRL depletion using a Phospho Explorer antibody array (Full Moon BioSystems, Sunnyvale, CA) and bioinformatics analysis. TIPRL depletion was confirmed to inactivate CaMK4, AKT1, AMPKα, and CREB1 (Figure S3A,B, Supporting Information). A schematic diagram based on the array data was used to illustrate the signaling pathway regulated by TIPRL (Figure S3C, Supporting Information). The CaMKK2‐CaMK4‐CREB signaling axis might contribute to maintaining the stemness and survival of lung CSCs.

### 2.2 TIPRL Binds CaMKK2, Which is also Upregulated in Lung CSCs

**Figure 2:**
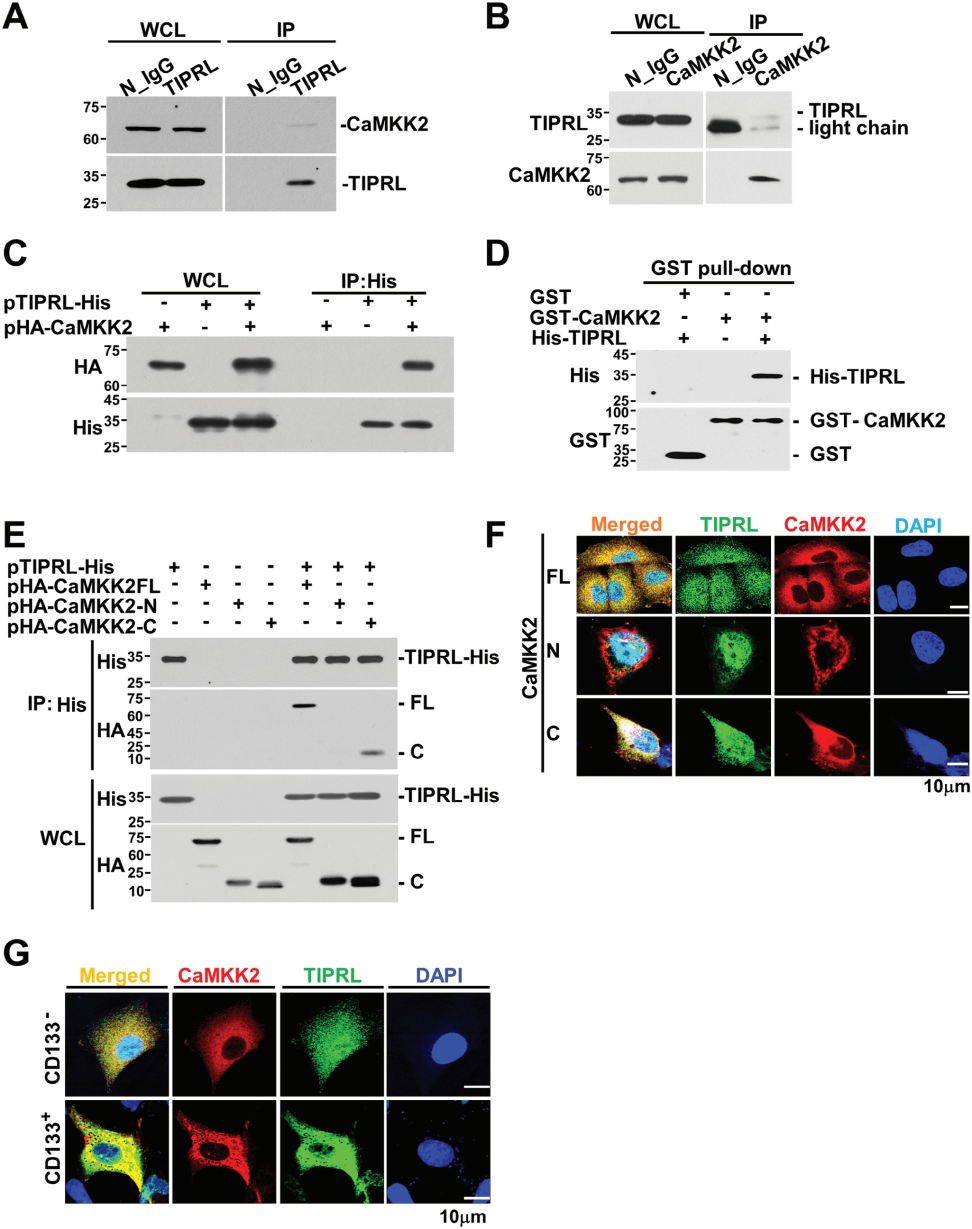
TIPRL binds CaMKK2. Immunoblotting for A) TIPRL and B) CaMKK2 immunoprecipitated from A549 cells; normal IgG was used as a negative control. C) IP assay of A549 cells transfected with pTIPRL‐His and pHA‐CaMKK2 plasmids. D) GST pull‐down assay of recombinant TIPRL and CaMKK2. E) IP assay and ICC F) results for ICC HEK293 cells co‐transfected with pTIPRL‐His and pHA‐CaMKK2 or deletion mutants. G) ICC analysis for CD133^+^ A549 cells using antibodies against CaMKK2 and TIPRL. TIPRL, target of rapamycin signaling pathway regulator; IP, immunoprecipitation; ICC, immunocytochemistry; PP2Ac, catalytic subunit of protein phosphatase 2A; WCL, whole cell lysate; DAPI, 4′,6‐Diamidino‐2‐Phenylindole.

To investigate the role of TIPRL as a negative regulator of the catalytic subunit of PP2A in the CaMKK2‐CaMK4‐CREB signaling axis, we conducted co‐immunoprecipitation assays to assess the interaction between CaMKK2 and TIPRL under physiological conditions in A549 cells. Additionally, we verified whether TIPRL blocks the interaction between CaMKK2 and PP2Ac, thereby preventing PP2Ac from inactivating CaMKK2. Immunoprecipitation using an endogenous antibody against TIPRL and CaMKK2 revealed that TIPRL binds to CaMKK2 (**Figure** **2**A,B, Supporting Information). Moreover, the binding of TIPRL to CaMKK2 was demonstrated using a co‐immunoprecipitation assay (after transfection with His‐tagged TIPRL and hemagglutinin‐tagged CaMKK2), as well as an in vitro GST pull‐down assay (Figure 2C,D).

To identify the binding regions of CaMKK2, we first generated deletion constructs of CaMKK2 (Figure S4A, Supporting Information). CaMKK2 constructs containing the C‐terminal region (amino acids 447–540) were precipitated using TIPRL (Figure 2E). Co‐immunostaining confirmed the co‐localization of TIPRL and the C‐terminal region of CaMKK2 (Figure 2F). We then examined the changes in the interaction of PP2Ac with CaMKK2 in siTIPRL‐transfected A549 cells. The interaction between CaMKK2 and PP2Ac was stronger after siTIPRL transfection (Figure S4B,C, Supporting Information), whereas the interaction was reduced after TIPRL overexpression (Figure S4D, Supporting Information). In addition, immunoprecipitation and immunostaining revealed PP2Ac bound to the C‐terminal region of CaMKK2 (Figure S4E,F, Supporting Information). Finally, we confirmed the interaction between TIPRL and CaMKK2 via co‐immunostaining of lung CSCs (Figure 2G). These results suggest that TIPRL blocks the interaction between CaMKK2 and PP2Ac, which may contribute to the maintenance of stemness and survival of lung CSCs.

To determine the role of CaMKK2 as a binding partner of TIPRL in lung CSCs, we evaluated its mRNA expression levels using qRT‐PCR. The mRNA level of CaMKK2 was higher in patients with SCLC and LUAD versus adjacent normal tissues (Figure S5A–C, Supporting Information); in CSCs isolated from several lung cancer cell lines (e.g., A549, H460, H1299, and H1793) versus non‐CSCs (Figure S5D, Supporting Information); and in CSCs freshly isolated from tissues of patients with lung cancer versus non‐CSCs (Figure S5E, Supporting Information). Using TCGA cohorts, we also confirmed that CaMKK2 expression was associated with the survival rate of patients with lung cancer, with a higher CaMKK2 expression associated with a decreased patient survival rate (Figure S5F, Supporting Information). To demonstrate the clinical significance of TIPRL and CaMKK2 in lung CSCs, we evaluated the expression of two proteins in CSCs freshly isolated from tissues of patients with lung cancer. TIPRL and CaMKK2 protein expression levels in lung CSCs were increased (Figure S5G, Supporting Information). Additionally, immunostaining of CaMKK2 and TIPRL in a similar tumor region stained strongly (Figure S5H, Supporting Information). Finally, the correlation between TIPRL and CaMKK2 expression was assessed among CSCs isolated from patients with lung cancer and in the data of patients with lung cancer from the TCGA cohort (Figure S5I,J, Supporting Information). Our findings suggest that the interaction between TIPRL and CaMKK2, which are both highly expressed in lung CSCs and bulk tumor cells, could be related to the poor prognosis of patients with lung cancer.

### 2.3 CaMKK2‐Induced Activation of CaMK4 Leads to the Phosphorylation of CREB and Maximum Transcriptional Activation of Target Genes in Lung Cancer

**Figure 3:**
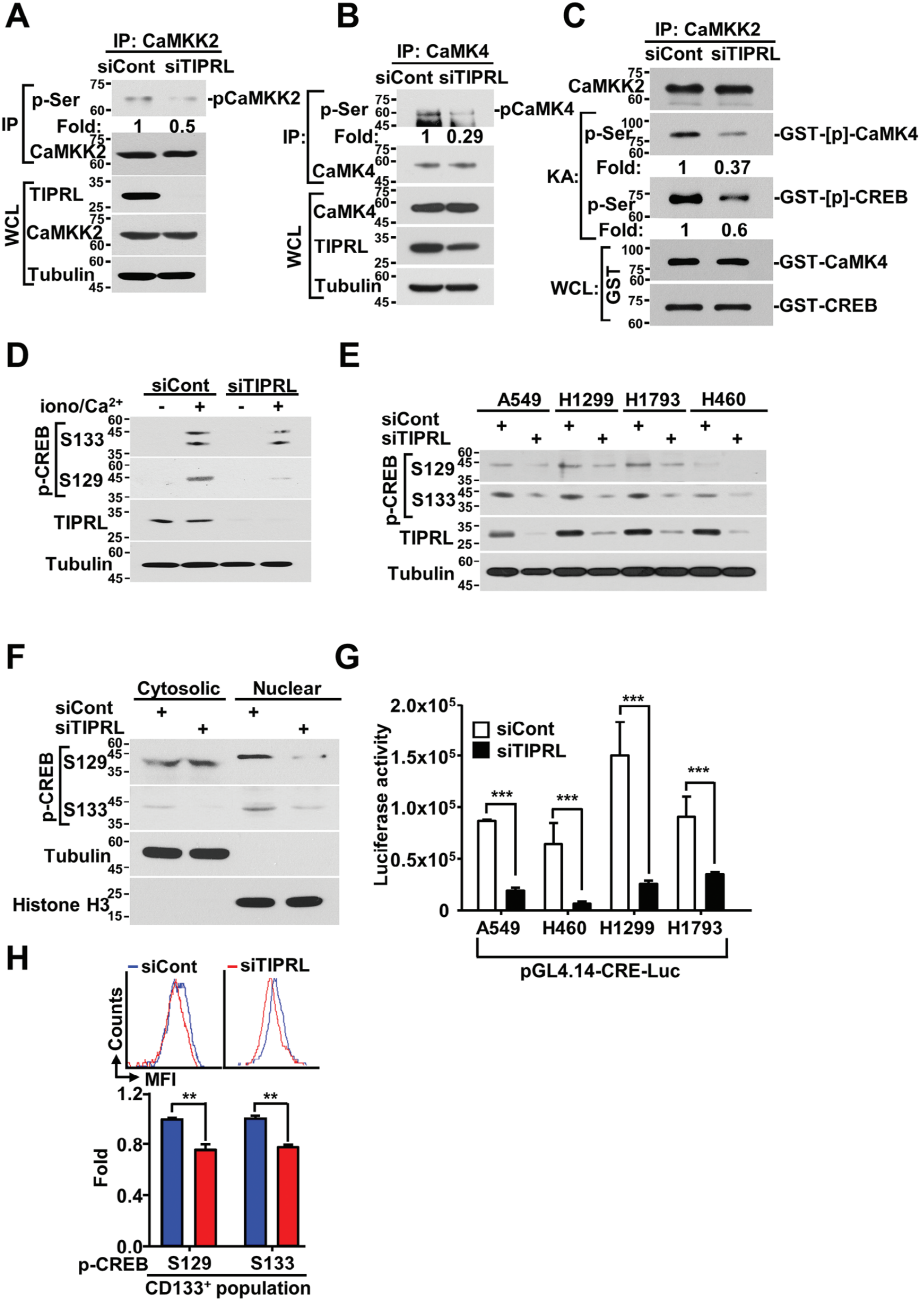
TIPRL depletion inactivates CaMKK2, downregulating the CaMKK2‐CaMK4‐CREB signaling pathway. Phosphorylation of A) CaMKK2 and B) CaMK4 immunoprecipitated from A549 cells transfected with the siRNA against *TIPRL* gene and control siRNA, analyzed using a phospho‐serine antibody. C) Phosphorylation of CaMK4 proteins by CaMKK2, as well as that of CREB by the activated CaMK4, in TIPRL‐depleted A549 cells, analyzed using an in vitro kinase assay with a phospho‐serine antibody. D) Immunoblotting of ionomycin‐ and CaCl_2_‐stimulated TIPRL‐depleted A549 cells using phospho‐CREB antibodies. E) Immunoblotting of A549, H1299, H1793, and H460 lung cancer cells transfected with an siRNA against TIPRL gene. F) Protein analysis of cytosolic and nuclear fractions of TIPRL‐depleted A549 cells using antibodies against tubulin (cytosolic marker) and histone H3 (nuclear marker). G) Dual‐Luciferase assay of the indicated lung cancer cells co‐transfected with a CRE‐driven luciferase reporter plasmid and siRNA against *TIPRL* gene. H) FACSCanto II analysis of CD133‐positive siTIPRL‐transfected A549 cells after staining with phospho‐CREB antibodies. TIPRL, target of rapamycin signaling pathway regulator; CRE, cAMP response element; CREB, cAMP response element‐binding protein; CaMKK2, calcium/calmodulin‐dependent protein kinase kinase 2; CaMK4, calcium/calmodulin‐dependent protein kinase 4.

To determine the role of TIPRL in the CaMKK2 signaling axis of lung CSCs, we first evaluated the alteration in CaMKK2 phosphorylation and its downstream kinase CaMK4 after TIPRL depletion via immunoprecipitation with antibodies against CaMKK2 and CaMK4 proteins. TIPRL depletion resulted in decreased CaMKK2 phosphorylation and, subsequently, CaMK4 phosphorylation (**Figure** **3**A,B). In addition, using an in vitro kinase assay, we confirmed that TIPRL depletion decreased the phosphorylation of CaMK4 and its downstream transcription factor CREB (Figure 3C).

CREB is a transcription factor that binds at the cAMP response element (CRE) of its target genes. CREB is phosphorylated at Ser133 by various receptor‐activated protein kinases, such as protein kinase A, mitogen‐activated protein kinases, and CaMK.^[^ ^21^, ^22^ ^]^ It remains unknown whether CaMKK2‐induced CaMK4 phosphorylates Ser133 or other CREB sites in lung cancer.

To identify the sites at which CREB is phosphorylated by CaMK4, we generated mutant CREBs in which serine residues at known phosphorylation sites, including Ser121, Ser129, Ser133, and Ser142/143, were changed to alanine. Mutant CREBs were first tested in a CRE‐driven reporter assay, and Ser129 was identified as the second site phosphorylated by CaMK4 (Figure S6A, Supporting Information). Maximum activation of CREB occurred when it was phosphorylated by CaMK4 at both Ser129 and Ser133. Moreover, we assessed the phosphorylation of one of the mutant CREBs through immunoprecipitation with a His antibody against CREB‐His and revealed that CaMK4 phosphorylated both Ser133 and Ser129 on CREB (Figure S6B, Supporting Information).

To determine the effects of TIPRL depletion on CREB phosphorylation, we stimulated siTIPRL‐transfected A549 cells with Ca^2+^ after 12 h of serum and Ca^2+^ starvation. TIPRL depletion decreased CREB phosphorylation at both Ser129 and Ser133 (Figure 3D). Under physiological conditions, TIPRL depletion decreased the phosphorylation of both CREB sites in several lung cancer cell lines (Figure 3E). Subsequently, decreased CREB phosphorylation after TIPRL depletion resulted in limited CREB translocation into the nucleus (Figure 3F). Furthermore, a CRE‐driven reporter assay revealed that the transcriptional activity of CREB decreased after TIPRL depletion in several lung cancer cell lines (Figure 3G). Finally, in lung CSCs, TIPRL knockdown decreased the phosphorylation of CREB at Ser129 and Ser133 (Figure 3H). In summary, TIPRL prolongs CaMKK2‐CaMK4‐CREB activation mediated by Ca^2+^ stimulation. This sustained activation may be crucial in maintaining the stemness and survival of lung CSCs.

To determine whether the reduction in CREB activity caused by TIPRL depletion resulted from the downregulation of CaMKK2, we evaluated the effect of CaMKK2 knockdown on CREB phosphorylation. As expected, there was reduced phosphorylation of CREB at Ser129 and Ser133 after CaMKK2 depletion. In contrast, CaMKK2 overexpression led to increased CREB activity (Figure S6C,D, Supporting Information). Additionally, a CRE‐driven reporter assay showed that CaMKK2 depletion in several lung cancer cell lines resulted in decreased CREB activity, whereas CaMKK2 overexpression increased it (Figure S6E,F, Supporting Information). These results indicate a direct link between TIPRL and decreased CaMKK2‐CaMK4‐CREB activity.

### 2.4 TIPRL Contributes to Stemness and Survival of Lung CSCs through the Maintenance of Mitochondrial Function

**Figure 4:**
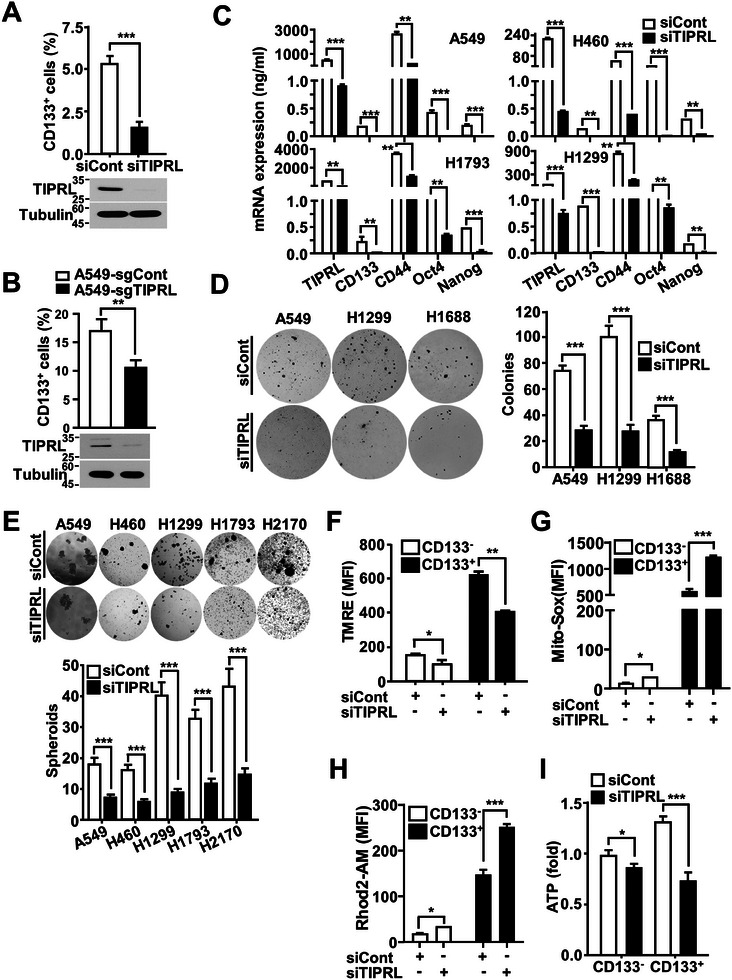
The CD133^+^ population of CSCs decreased significantly after TIPRL depletion. FACSCanto II analysis of CD133^+^ populations in A) siTIPRL‐transfected A549 cells and B) the stable TIPRL‐depleted A549‐sgTIPRL cell line. C) qRT‐PCR analysis to assess alterations in stemness‐related gene expression caused by TIPRL depletion in lung cancer cells. D) Soft agar and E) sphere‐formation assays of lung cancer cells transfected with an siRNA against TIPRL and control siRNA for 72 h; results show the number of colonies and spheroids generated per 10 000 counted after 3 and 2 weeks, respectively. Measurement of F) the mitochondrial membrane potential (TMRE staining), G) reactive oxygen species (ROS) levels (Mito‐SOX staining), H) Ca^2+^ levels (Rhod2‐AM staining), and I) ATP levels in CD133^–^ and CD133^+^ siTIPRL‐transfected A549 cells. TIPRL, target of rapamycin signaling pathway regulator; TMRE, tetramethylrhodamine; ROS, reactive oxygen species; CD, cluster of differentiation; siRNA, short interfering RNA; CaMKK2, calcium/calmodulin‐dependent protein kinase kinase 2.

To investigate the role of TIPRL in CSCs, TIPRL was depleted using siRNA and single guide RNA (sgRNA) molecules. As expected, the CSC population percentage was significantly decreased in siTIPRL‐transfected cells and the stable TIPRL‐depleted A549‐sgTIPRL cell line versus controls (**Figure** **4**A,B). In these cells, a decreased overall expression of stemness‐related genes was noted (Figure 4C). Moreover, an anchorage‐independent growth assay revealed that TIPRL‐depleted cells displayed a significant reduction in sphere and colony‐formation versus controls (Figure 4D,E). TIPRL depletion in several lung cancer cell lines also reduced migration and wound‐healing activity versus controls (Figure S7A,B, Supporting Information). A rescue experiment using a TIPRL‐expressing lentivirus effectively restored the TIPRL knockdown‐induced reduction in CSCs (CD133^+^ cells) and the migrating population (Figure S7C–E, Supporting Information).

Mitochondrial activity regulates stemness and pluripotency, which are properties of CSCs.^[^ ^23^, ^24^, ^25^, ^26^ ^]^ To assess whether inhibition of the CaMKK2‐CaMK4‐CREB axis upon TIPRL depletion in lung CSCs led to the disruption of mitochondrial function, we measured changes in membrane potential, mitochondrial reactive oxygen species (ROS) levels, and calcium levels using tetramethylrhodamine (TMRE), Mito‐SOX, and Rhod2‐AM dyes, respectively, in siTIPRL‐depleted cells.

TIPRL depletion in lung CSCs and non‐CSCs decreased the mitochondrial membrane potential but increased ROS and calcium levels (Figure 4F–H). CD133^+^ cells had notably higher ATP levels than non‐CSCs, but these decreased after TIPRL depletion (Figure 4I; Figure S7F, Supporting Information). Taken together, these results suggested that TIPRL plays a critical role in maintaining lung CSCs properties and mitochondrial function.

### 2.5 TIPRL Knockdown Leads to Downregulation of CREB Target Genes (Bcl2, HMG20A) through Inactivating the CaMKK2 Signaling Axis

**Figure 5:**
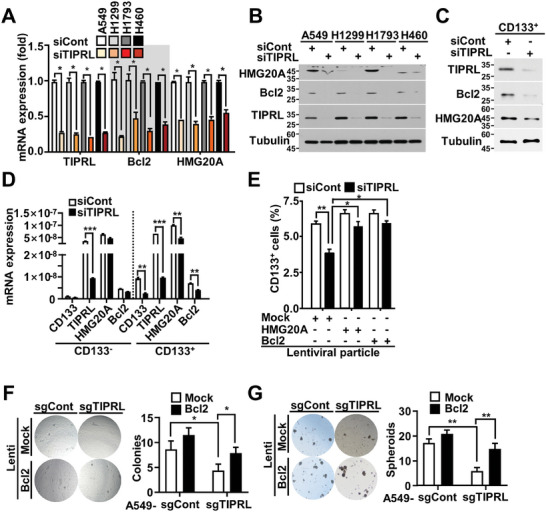
TIPRL promotes the expression of CREB target genes, including Bcl2 and HMG20A, in lung CSCs. A) qRT‐PCR and B) immunoblotting analysis of CREB target gene expression in lung cancer cells transfected with siTIPRL or siCont. C) Immunoblotting analysis of the expression of Bcl2 and HMG20A in CD133^+^ TIPRL‐depleted A549 cells. D) qRT‐PCR analysis of CREB target gene expression in CD133^–^ and CD133^+^ cells lung cancer cell lines. E) FACSCanto II analysis to identify the CD133^+^ population of CSCs in TIPRL‐depleted A549 cells infected with HMG20A, Bcl2, or mock lentivirus. F) Soft agar and G) sphere‐formation assays; the number of colonies and spheroids were counted after 2 weeks. TIPRL, target of rapamycin signaling pathway regulator; ROS, reactive oxygen species; CREB, cAMP response element‐binding protein; CD, cluster of differentiation; qRT‐PCR, quantitative real‐time reverse‐transcription PCR; CSCs, cancer stem cells.

We investigated the molecular mechanisms underlying the crosstalk between the CaMKK2 signaling pathway and lung CSCs. Liquid chromatography with tandem mass spectrometry analysis was done to identify the proteins affected by CREB inactivation in TIPRL‐depleted CSCs (Figure S8A–D, Supporting Information). Several potential target genes were identified; we hypothesize that these are modulated by the TIPRL‐CaMKK2 signaling axis. Additionally, these genes are postulated to play a pivotal role in maintaining stemness and enhancing survival in lung CSCs. To test this hypothesis, we first investigated if the TIPRL‐CaMKK2 signaling pathway regulates these genes. TIPRL depletion significantly reduced the mRNA and protein levels of Bcl2 and HMG20A, among various potential target genes, in several lung cancer cell lines (**Figure** **5**A,B). However, the decrease in Bcl2 and HMG20A expression caused by TIPRL depletion was rescued after infecting the cells with TIPRL‐expressing lentiviral particles (Figure S8E, Supporting Information). TIPRL depletion in CD133^+^ cells isolated from lung cancer cells also decreased the expression of Bcl2 and HMG20A (Figure 5C,D).

We investigated whether CREB, a transcription factor in the CaMKK2‐CaMK4‐CREB axis, transcriptionally regulates HMG20A and Bcl2. A bioinformatics analysis revealed that the human Bcl2 and HMG20A promoters contained CREB‐binding sites (Bcl2: TGACGTTA and HMG20A: CGTCA) located 89 and −1790 bp upstream from the transcriptional start site, respectively (Figure S8F, Supporting Information). A CREB‐binding reporter assay revealed that the promoter activity of Bcl2 and HMG20A was increased significantly alongside increasing CREB expression (Figure S8G, Supporting Information) but was decreased after TIPRL depletion versus controls, indicating that CREB is a component of the CaMKK2 signaling axis regulated by TIPRL (Figure S9A, Supporting Information). Cut‐and‐run assays confirmed that CREB directly binds to the Bcl2 and HMG20A promoter regions containing a CREB‐binding site (Figure S9B, Supporting Information). Meanwhile, rescue experiments with Bcl2‐ and HMG20A‐expressing lentiviral particles were conducted to determine whether the decrease in tumor progression caused by TIPRL depletion was directly linked to the expression of Bcl2 and HMG20A in TIPRL‐depleted cells. The lentiviral infection led to the overexpression of Bcl2 and HMG20A, which ameliorated the reduction in cell proliferation and CD133^+^ cell population caused by TIPRL depletion (Figure S9C, Supporting Information; Figure 5E) and enhanced anchorage‐independent growth in TIPRL‐depleted A549 and control cells (Figure 5F,G). In addition, the membrane potential, ROS levels, and calcium levels were also restored by the overexpression of Bcl2 and HMG20A (Figure S9D–F, Supporting Information). In short, TIPRL contributes to the activation of the CaKK2‐CaMK4‐CREB signaling axis, leading to a sustained expression of CREB target genes such as Bcl2 and HMG20A. In turn, these contribute to the stemness and tumorigenic ability of CSCs, as well as the maintenance of mitochondrial function.

### 2.6 TIPRL Depletion Enhances the Sensitivity of Lung CSCs to Afatinib and Impairs Tumor Growth and Metastasis through CaMKK2 Signaling Inhibition In Vitro and In Vivo

**Figure 6:**
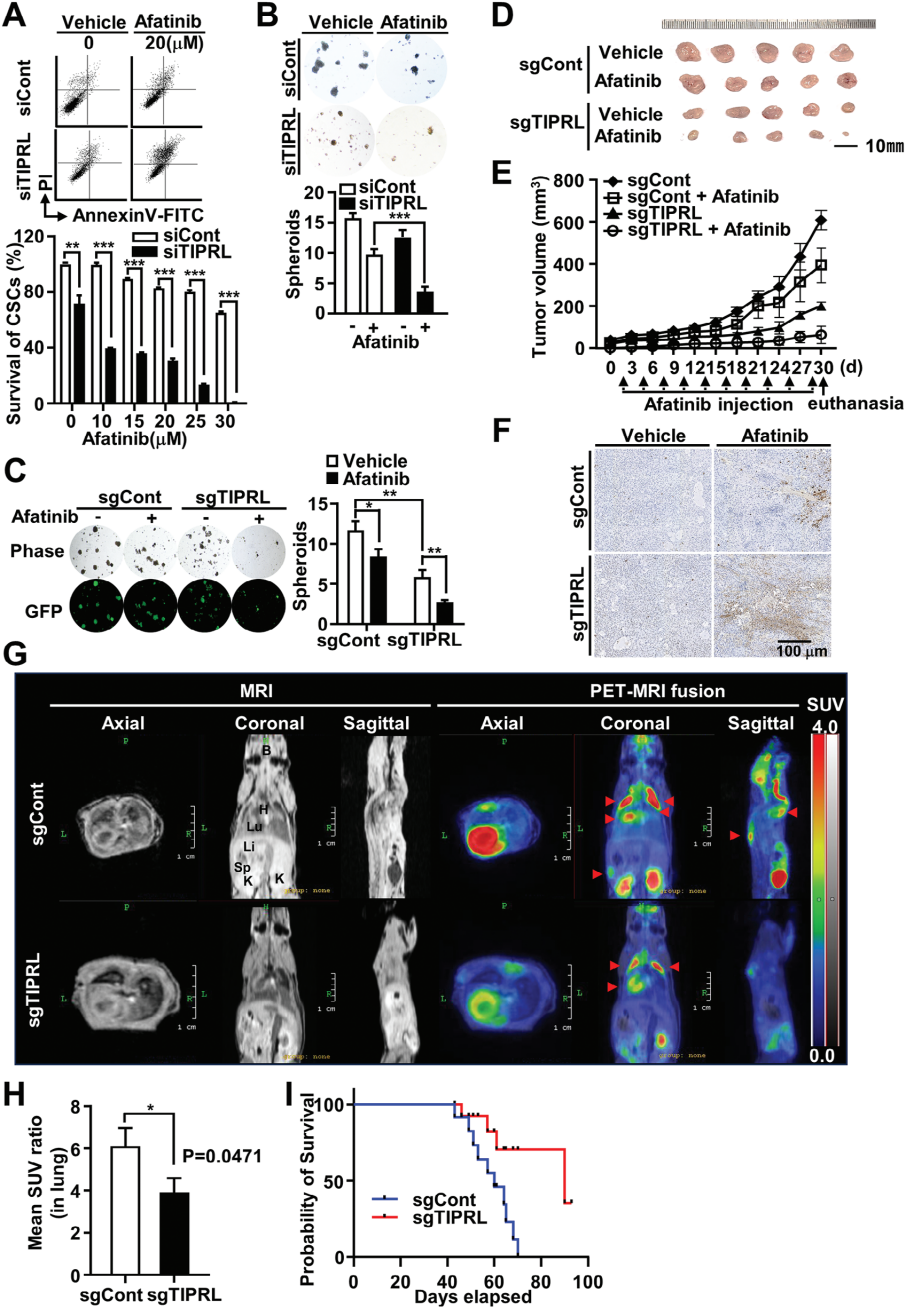
TIPRL depletion enhances cell death induced by afatinib. A) Cell death of lung CSCs in CD133^–^ and CD133^+^ TIPRL‐depleted A549 cells treated with the indicated dose of afatinib for 24 h, measured using FACSCanto II after staining with Annexin V‐FITC/PI. Sphere‐formation assay of B) siTIPRL‐transfected A549 cells and C) the A549‐sgTIPRL cell line treated with 20 µm afatinib for 24 h and incubated with 20 ng mL^−1^ epidermal growth factor and 10 ng mL^−1^ basic fibroblast growth factor for 3 weeks. A549‐sgTIPRL cells express to the EGFP supported as a selection tool for gene editing using a CRISPR/Cas9. The generated spheroids were analyzed using fluorescence microscopy to detect the GFP‐expressing cells. D) Images of tumors in TIPRL‐depleted mice subjected to the following treatments: sgCont, sgCont+afatinib, sgTIPRL, and sgTIPRL+afatinib; n = 5 each treatment group. E) Mean tumor volumes recorded at 3‐day intervals ± standard error. F) Terminal deoxynucleotidyl transferase‐mediated deoxyuridine triphosphate nick‐end labeling staining of tumor samples from severe combined immunodeficiency mice intrathoracically injected with CD133^+^ A549‐sgCont and sgTIPRL cells to determine the effects on lung metastasis. G) Analysis of tumor growth and metastasis after 7 weeks post‐injection using a nano positron emission tomography‐magnetic resonance imaging system with ^18^F‐FDG. H) Mean SUV ratio in the lungs. I) Survival curves for animals from the two groups. TIPRL, target of rapamycin signaling pathway regulator; CD, cluster of differentiation; CSCs, cancer stem cells; FITC, fluorescein isothiocyanate; PI, propidium iodide; SUV, standard uptake value; PET, positron emission tomography; MRI, magnetic resonance imaging; ^18^F‐FDG, ^18^F‐fludeoxyglucose.

To investigate the role of increased CaMKK2/CaMK4/CREB signaling activity in lung CSC survival, we conducted cell death‐inducing experiments by treating cells with afatinib (an EGFR tyrosine kinase inhibitor) alone or in combination with TIPRL depletion. CSC subpopulations are more resistant to afatinib than non‐CSC cells.^[^ ^27^, ^28^ ^]^ TIPRL depletion plus afatinib treatment resulted in a significant increase in CD133^+^ cell death, as seen on flow cytometry with Annexin V‐FITC/propidium iodide staining (**Figure** **6**A; Figure S10A, Supporting Information). Additionally, combination treatment also led to an increase in CSC death, as seen on the Cell Counting Kit‐8 assay (Figure S10B,C, Supporting Information). To confirm that the effects of the combination treatment were due to apoptosis, we measured the sub‐G1 phase DNA content. A significant increase was seen in the sub‐G1 hypodiploid population in CSCs given combination therapy versus afatinib alone (Figure S10D,E, Supporting Information). Moreover, increased levels of cleaved procaspase‐3 and poly‐(adenosine diphosphate ribose) polymerase were observed in TIPRL‐depleted cells treated with low‐dose afatinib versus control cells (Figure S10F,G, Supporting Information). This result demonstrated that knockdown of TIPRL sensitizes CSCs to afatinib‐induced cell death via inhibition of the CaMKK2 signaling axis, which is a consequence of TIPRL depletion as shown in Figure 3. Next, the effect of the combination treatment on CSC‐mediated tumorigenesis was observed using an anchorage‐independent growth assay. Colony‐formation was significantly reduced in cells treated with combination therapy versus control cells (Figure S10H,I, Supporting Information). Furthermore, in sphere‐formation assays, cells treated with combination therapy also exhibited reduced sphere‐formation versus control cells (Figure 6B,C).

To evaluate the in vivo effects of TIPRL depletion, a mouse xenograft model was generated by injecting CD133^+^ cells sorted from A549‐sgTIPRL or A549‐sgCont cells. CD133^+^ A549‐sgTIPRL cells had a greatly reduced tumor volume versus A549‐sgCont cells (Figure 6D,E). Furthermore, terminal deoxynucleotidyl transferase‐mediated deoxy‐uridine triphosphate nick‐end labeling revealed that this effect was due to increased apoptosis in the sgTIPRL+afatinib‐treated tumors (Figure 6F). To confirm that these effects were due to a decreased TIPRL expression, the TIPRL expression in xenograft tumors was analyzed via immunostaining. TIPRL expression decreased in xenograft tumors generated by A549‐sgTIPRL cells compared to that in tumors generated by A549‐sgCont cells (Figure S10J, Supporting Information).

Lung cancer often metastasizes to the brain, bones, and the liver.^[^ ^29^, ^30^ ^]^ We evaluated the effect of TIPRL depletion on metastasis in an orthotopic xenograft model generated by injecting CSCs into the lungs of mice. The positron emission tomography‐magnetic resonance imaging (PET‐MRI) scan revealed significantly reduced local invasion and metastasis in the TIPRL‐depleted group compared with those in the control group. Specifically, injecting A549‐sgCont‐CD133^+^ cells into the left lung resulted in liver metastasis in 50% (5/10) of the mice, whereas injecting A549‐sgTIPRL‐CD133^+^ cells resulted in liver metastasis in 20% (2/10) (Figure 6G,H). Finally, the effect of TIPRL depletion on survival rate was examined. The survival rate was higher in mice that received TIPRL‐depleted cells versus controls, likely due to the decreased tumor frequency observed in the TIPRL‐depleted group (Figure 6I). These in vivo results suggest that TIPRL regulates the CaMKK2 signaling axis, which contributes to anticancer drug resistance and stemness in lung CSCs.

### 2.7 TIPRL is Transcriptionally Activated by CREB in Lung CSCs

**Figure 7:**
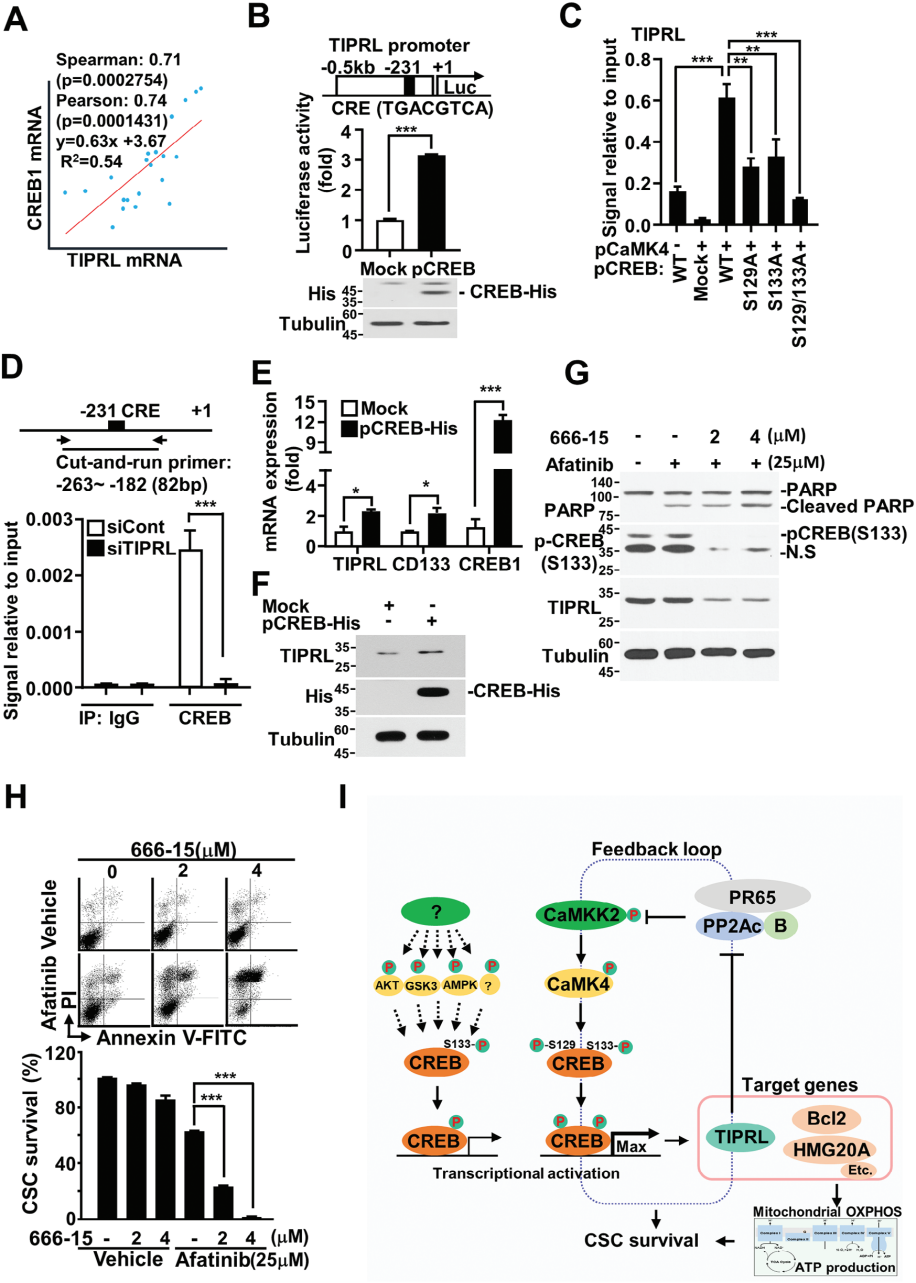
TIPRL is transcriptionally activated by CREB, leading to sustained activation of the CaMKK2 signaling axis via a positive feedback loop. A) Comparison of TIPRL and CREB1 expression in the TCGA lung cancer cohort. B) TIPRL promoter activity in HEK 293 cells co‐transfected with TIPRL promoter‐driven luciferase reporter plasmids and His‐tagged‐CREB‐overexpressing plasmids. C) qRT‐PCR results showing the association of CREB with the TIPRL promoter in HEK 293 cells co‐transfected with pCaMK4‐flag and wild‐type or mutant forms of CREB‐His (CREB S129A, S133A, and S129/133A), followed by a cut‐and‐run assay using a His antibody. D) Results of a cut‐and‐run assay of TIPRL‐depleted A549 cells using a CREB antibody or normal IgG. qRT‐PCR primer location and CRE‐binding site on the promoter of the *TIPRL* gene. E) mRNA expression, determined by qRT‐PCR with primers against *TIPRL*, *CD133*, and *CREB1* genes, and F) protein levels, determined through immunoblotting with antibodies against TIPRL and His, in HEK293 cells transfected with CREB1‐overexpressing plasmids or mock. Analysis of G) alterations in protein levels by immunoblotting with the indicated antibodies and H) cell death using FACSCanto after staining with Annexin V‐FITC/PI in A549 cells treated with the CREB inhibitor 666‐15 and afatinib for 24 h. I) Schematic illustration of the expression and mechanism of action of TIPRL and the CaMKK2‐CaMK4‐CREB axis in lung CSCs. In CSCs, TIPRL, Bcl2, and HMG20A were significantly upregulated by CREB, via activation of the CaMKK2‐CaMK4‐CREB signaling pathway. TIPRL, as a positive feedback regulator, activates the CaMKK2 axis, while elevated Bcl2 maintains the mitochondrial membrane potential and contributes to ATP production by the mitochondrial OXPHOS in lung CSCs. Thereby, the functions of the TIPRL‐CaMKK2 signaling loop seem to be critical for maintaining the self‐renewal and tumorigenic abilities of lung CSCs. CaMKK2, calcium/calmodulin‐dependent protein kinase kinase 2; TIPRL, target of rapamycin signaling pathway regulator; CSCs, cancer stem cells; OXPHOS, oxidation phosphorylation; CREB, cAMP response element‐binding protein; CD, cluster of differentiation; CaMK4, calcium/calmodulin‐dependent protein kinase 4; qRT‐PCR, quantitative real‐time reverse‐transcription PCR; FITC, fluorescein isothiocyanate; PI, propidium iodide; TCGA, The Cancer Genome Atlas.

The regulatory mechanism of TIPRL expression in cancer cells remains unknown. A bioinformatics analysis was performed to investigate the transcription factors which bind to the TIPRL gene promoter. A CREB‐binding site (TGACGTCA) was identified −231 bp upstream of the TIPRL transcriptional start site. In a TCGA cohort study, a positive correlation was found between TIPRL and CREB1 mRNA expression (**Figure** **7**A). Moreover, the activity of the TIPRL promoter (−500 to +20) was significantly increased with CREB overexpression, indicating that TIPRL is a transcriptional target of CREB (Figure 7B). A cut‐and‐run assay in CREB mutant‐overexpressing cells showed that CREB binds directly to the TIPRL promoter containing a CREB‐binding site (Figure 7C). Furthermore, we found that the binding of CREB to the TIPRL promoter was considerably decreased after TIPRL depletion, suggesting that TIPRL plays a critical role in regulating the CaMKK2 signaling axis and is a transcriptional target of CREB regulated by the signaling axis at the same time (Figure 7D). Additionally, TIPRL protein and mRNA levels were increased alongside CREB overexpression in HEK293 cells with lower TIPRL expression (Figure 7E,F). By contrast, the combined treatment of afatinib and 665‐15 (CREB inhibitor) in A549 cells considerably downregulated TIPRL by reducing CREB phosphorylation compared with that observed in vehicle‐ or afatinib‐treated control cells and then induced apoptosis (Figure 7G). Finally, flow cytometry using Annexin V‐FITC/propidium iodide staining revealed a significant increase in CD133^+^ CSC death following combination treatment with 666‐15 and afatinib (Figure 7H). In summary, TIPRL induces sustained activation of the CaMKK2‐CaMK4‐CREB axis. CREB activation upregulates the expression of TIPRL, Bcl2, and HMG20A to maintain the stemness and survival of CSCs, thus promoting tumorigenesis in lung cancer.

## 3 Discussion

Our study revealed that TIPRL is upregulated in lung CSCs and identified CaMKK2 as its novel binding partner. TIPRL binds to the C‐terminal region of CaMKK2, resulting in its sustained activation. CaMKK2 is a key regulator of cellular and whole‐body energy metabolism which protects cells from ferroptosis in melanoma,^[^ ^31^ ^]^ regulates mitochondrial function,^[^ ^32^ ^]^ and is upregulated in hepatocellular carcinoma and prostate cancer.^[^ ^33^, ^34^ ^]^ Previous studies on CSCs have reported the importance of mitochondrial function in the alteration of CSC fate.^[^ ^35^, ^36^, ^37^, ^38^ ^]^ In present study, we found that CaMKK2 was also upregulated in lung CSCs and bulk tumor cells, and TIPRL depletion allowed PP2Ac to bind to the C‐terminal region of CaMKK2, leading to inactivation of the CaMKK2 signaling pathway. We showed that CaMK4, which is activated by CaMKK2, phosphorylates CREB at Ser129 and Ser133 to fully activate it in lung CSCs. CREB is a ubiquitous transcription factor that regulates cell proliferation and survival.^[^ ^39^ ^]^ It is regulated by several distinct pathways that can become activated in cancer cells^[^ ^40^, ^41^, ^42^ ^]^ and is activated through the phosphorylation of Ser133 in response to cAMP, Ca^2+^, and other stimuli.^[^ ^43^ ^]^ CREB Ser133 phosphorylation was found to be essential, but not sufficient, to ensure CRE‐related gene transcription.^[^ ^44^, ^45^, ^46^, ^47^ ^]^ In the present study, phosphorylation at Ser129 and Ser133 positively affected CREB transcriptional activity, whereas that at Ser111 and Ser121 completely blocked CREB‐related gene expression. Using CREB mutants (serine residues with well‐known phosphorylation sites for alanine, including Ser121, Ser129, Ser133, and Ser142/143), we investigated the CREB sites phosphorylated by CaMK4 and showed that phosphorylation at Ser129 and Ser133 is required for the maximum activation of CREB by the CaMKK2/CaMK4 signaling pathway in lung CSCs. TIPRL depletion resulted in a decrease in the phosphorylation of CREB Ser129 and Ser133, thereby compromising the stemness and survival of lung CSCs.

CREB plays a role in various cellular processes such as proliferation, survival, and glucose metabolism, depending on the gene expression in cancer cells.^[^ ^48^ ^]^ The target genes of CREB are differentially expressed according to the type of cancer and the upstream kinases that regulate them.^[^ ^49^ ^]^ *Bcl2* is a known CREB target gene that encodes a protein that mediates the pro‐survival effects of CREB and acts as an inhibitory binding partner of Bax, forming a channel or other structure in the mitochondrial outer membrane.^[^ ^50^, ^51^ ^]^ In this study, we identified *Bcl2* and *HMG20A* as CREB target genes in lung CSCs and showed that TIPRL depletion rescued the reduction in stemness and survival of lung CSCs through the overexpression of *Bcl2*. Therefore, a reduction in Bcl2 levels can lead to apoptotic cell death and mitochondrial dysfunction. We also showed that TIPRL depletion resulted in mitochondrial dysfunction, including decreased mitochondrial membrane potential and increased ROS and calcium levels. This can be attributed to a decrease in Bcl2 protein and the formation of a Bax channel in the mitochondrial membrane. Furthermore, the collapse of the CaMKK2 signaling pathway after TIPRL depletion led to significantly reduced ATP levels in CSCs, with only a marginal decrease in non‐CSCs. A rescue experiment involving Bcl2 overexpression in TIPRL‐depleted cells confirmed that TIPRL tightly regulates mitochondrial function via CREB activation.

Combination treatment with afatinib and TIPRL depletion was able to induce apoptosis in lung CSCs, thus reducing their tumorigenic ability. TIPRL depletion caused a decrease in the expression of HMG20A, a novel target gene of CREB in lung CSCs. HMG20A is a high‐mobility group domain‐containing protein which is homologous to HMG20B.^[^ ^52^ ^]^ It regulates inflammatory responses and the epithelial‐mesenchymal transition, as well as enhances DNA damage repair.^[^ ^52^, ^53^, ^54^, ^55^ ^]^ Therefore, TIPRL‐induced reduction of HMG20A in lung CSCs probably abolishes resistance to the EGFR inhibitor afatinib. Lastly, TIPRL depletion combined with an anticancer drug could also be a promising approach against anticancer drug resistance, recurrence, and metastasis.

In liver and lung cancers, TIPRL is upregulated and plays a role as a tumor progressor.^[^ ^56^, ^57^, ^58^ ^]^ However, the mechanism that regulates TIPRL gene expression remains unknown. In this study, we found that CREB directly binds to the TIPRL promoter. In turn, increased TIPRL maintains sustained activation of the CaMKK2‐CaMK4‐CREB signaling axis by binding to the C‐terminal region of CaMKK2. CREB acts as a positive feedback controller by binding to the promoter of TIPRL and stimulating its transcription. This CREB‐TIPRL positive feedback regulation loop contributes to the maintenance of stemness and survival of lung CSCs (Figure 7I).

## 4 Conclusion

TIPRL is a promising therapeutic target for lung cancer, particularly for CSCs. Our study elucidates the molecular mechanisms underlying the crosstalk between the CaMKK2 signaling pathway and CSCs in lung cancer, which can aid in the development of novel treatment strategies.

## 5 Experimental Section

### Cell Culture and Antibodies

Human lung cancer (A549, H23, and H1793 for LUAD; H2170 and Calu‐1 for LUSC; H1299 and H460 for large cell carcinoma; H1688 and H69 for SCLC) and normal epithelial (HEL299, LL24, and Beas‐2B) cell lines were obtained from the Korean cell Line Bank (Seoul, South Korea). The lung cancer cell lines represent different types and subtypes of lung cancer, including non‐small cell lung cancer (NSCLC) and small cell lung cancer (SCLC). The histological type and genetic alteration of all cell lines used are presented in Table S1 (Supporting Information). This diversity was selected to allow for a broader understanding of how different lung cancer subtypes and genetic background respond to TIRPL and exhibit cancer stem cell characteristics. A549, H460, H1299, and H1793 cells were cultured in Dulbecco's modified Eagle's medium (DMEM)/F12 supplemented with 4 mg mL^−1^ bovine serum albumin, 10 ng mL^−1^ basic fibroblast growth factor, 20 ng mL^−1^ epidermal growth factor, 1× B27, 2 µg mL^−1^ heparin, and 25 mg mL^−1^ insulin, to obtain spheroids. All cell cultures were carried out at 37 °C, in a humidified incubator containing 5% CO_2_. All antibodies used are listed in Table S2 (Supporting Information). Briefly, antibodies against the following proteins were used: poly(adenosine diphosphate ribose) polymerase, caspase‐3, CaMKK2, and Histone H3 (Cell Signaling Technology, Danvers, MA, USA); CD133, including those conjugated to magnetic beads, allophycocyanin and CD44‐FITC (Miltenyi Biotec, Bergisch Gladbach, Germany); TIPRL (Bethyl Laboratories, Montgomery, TX, USA); PP2Ac, tubulin, CaMKK2, CREB, p‐CREB (S133), GST, and mouse and rabbit IgG‐HRP (Santa Cruz Biotechnology, Santa Cruz, CA, USA); hemagglutinin epitope (Roche, Nutley, NJ, USA); and p‐CREB (S129) (Thermo Fisher Scientific, Waltham, MA, USA). The mouse monoclonal anti‐His tag antibody was from Qiagen (Hilden, Germany).

### Tissue Collection and Cell Isolation from Patients with Lung Cancer

Human tissues were obtained from patients with lung cancer who participated in this study and provided written informed consent. All experimental protocols were approved by the Institutional Boards of Asan Medical Center and the University of Ulsan College of Medicine (approval nos. 2020‐1117 and 2021‐1264). Human lung tissues were obtained from the Asan Bio Resource Center, Korea Biobank Network [2021‐13(230)], from 11 patients (age range, 30–83 years) who underwent surgery for non‐small cell lung cancer. In the 11 tissue pairs, cells from each tissue sample were sorted as CD133^+^ or CD133^–^, and their total RNA was extracted. Patient case descriptions are presented in Table S3 (Supporting Information).

### Transfection, Transduction, and Stable Cell Line Construction

For *TIPRL* knockdown, siRNAs (5′‐CCTAATGAAATATCCCAGTAT‐3′) were constructed based on the *TIPRL* sequence, which exhibited >80% TIPRL knockdown efficiency. To generate a stable *TIPRL* knockdown, an sgRNA sequence targeting the *TIPRL* gene was subcloned into Tet‐On 3G all‐in sgRNA‐Cas9 plasmids (ToolGen, Seoul, South Korea), which were used to transfect A549 cells. For knockout validation, genomic DNA from the selected clones was sequenced after amplifying a nearby DNA sequence complementary to the *TIPRL* sgRNA. The optimal scoring guide target site, GCCCGAAGCAGAAATCCCGG[TGG] (PAM sites indicated in brackets), was selected for CRISPR gene editing via Cas9. The transfected cells were selected in a medium containing 2 µg mL^−1^ puromycin (Thermo Fisher Scientific) and then maintained in a medium containing 0.5 µg mL^−1^ puromycin. For *TIPRL* overexpression, A549 cells were transfected with 4 µg of pCGN‐HA or pCGN‐HA‐TIPRL using the Lipofectamine LTX reagent (Thermo Fisher Scientific). For the *TIPRL* rescue experiment, A549‐sgCont and A549‐sgTIPRL cells were infected with lentiviral particles expressing *TIPRL* or the control. *TIPRL* was subcloned into pLVX‐zsGreen‐N1 (TaKaRa Bio, Shiga, Japan) from pCGN‐HA‐TIPRL. To produce the recombinant lentivirus, pLVX‐TIPRL was co‐transfected with MISSION Lentiviral Packaging Mix (Sigma‐Aldrich, MA, USA) into HEK 293FT cells (Thermo Fisher Scientific). Viral particles were collected by carefully removing the medium at 48 h post‐transfection.

### Fluorescence‐Activated Cell Sorting, Magnetic‐Activated Cell Sorting, and Flow Cytometry

A549, H460, H1299, and H1793 human lung cancer cell aggregates were dissociated into single cells, washed with PBS, and stained with fluorescent antibodies specific for CD133/1‐allophycocyanin and CD44‐FITC (Miltenyi Biotec). Mouse IgG1 conjugated to allophycocyanin was used as the isotype control. The cells were sorted using a BD FACSAria flow cytometer (BD Biosciences, Franklin lakes, NJ, USA). Magnetic cell separation of the tumor cell populations was performed using microbeads conjugated with CD133/1 (AC133, mouse IgG, Cell Isolation Kit; Miltenyi Biotec). The magnetic separation step was repeated twice, using a positive selection column (LS column) once and a negative selection column (LD column) after, following which the eluted cells were applied to a new positive selection column. After magnetic sorting, the cell viability was assessed using a Trypan Blue exclusion assay. Sorting quality in both CD133^+^ and CD133‐depleted cell populations was assessed by flow cytometry using an antibody against CD133/2 (293C3‐phycoerythrin; Miltenyi Biotec).

### Phospho‐Specific Protein Antibody Array Assay

After adding 75 µL of labeling buffer to the protein sample (50 µg), the sample was treated with 3 µL of the 10 µg µL^−1^ biotin/DMF solution. The antibody microarray slide (Full Moon Biosystems) was treated with 30 mL of blocking solution in a Petri dish, incubated on a shaker at 60 rpm for 30 min at room temperature, and washed with distilled water. The labeled sample was mixed with 6 mL of coupling solution. The blocked array slide was incubated with coupling mixture on a shaker at 60 rpm for 2 h at room temperature in a coupling dish. Then, 30 µL of 0.5 mg mL^−1^ Cy3‐streptavidin (GE Healthcare, Chalfont St. Giles, UK) was mixed in 30 mL of detection buffer. The coupled array slide was treated with detection mixture in a Petri dish on a shaker at 60 rpm for 20 min at room temperature. The slide scanning was performed using a GenePix 4100A scanner (Axon Instrument, Sunnyvale, CA, USA). The slides were scanned at 10 µm resolution, optimal laser power, and PMT. After obtaining the scan image, they were grided and quantified using GenePix 7.0 Software (Axon Instrument). The protein information was annotated using UniProt DB. The phosphorylation ratio was calculated using the following formula:

(1) $$phophorylation\;ratio\;=\frac{\;phospho_{experiment}}{unphospho_{experiment}\;}/\frac{phospho_{control}}{unphospho_{control}}$$

Data mining and graphic visualization were performed using ExDEGA (Ebiogen Inc., Seoul, South Korea).

### In Vitro Cell Death Assays

Unsorted or magnetically sorted CD133^+^ and CD133^–^ A549 lung cancer cells were cultured in DMEM/F12 supplemented with 20 ng mL^−1^ epidermal growth factor and 10 ng mL^−1^ basic fibroblast growth factor. The cells were treated with afatinib at the indicated doses for 24 h (Selleck Chemicals, Huston, TX, USA). Similarly, the spheroids were cultured in the presence or absence of afatinib in DMEM/F12 supplemented with epidermal growth factor and basic fibroblast growth factor. The viability of unsorted, CD133^+^, and CD133^–^ cells was evaluated using the Annexin V‐FITC/PI Staining Kit (BD Biosciences) or CCK‐8 Assay Kit (Dojindo, Kumamoto, Japan). The effect of the spheroid culture system on sphere‐formation was evaluated using colony counting and microscopy.

### Immunohistochemistry of Tissue Samples

Patient tissues were fixed overnight with 10% neutral‐buffered formalin, embedded in paraffin, and processed into 5‐µm‐thick sections. The sections were deparaffinized in xylene, followed by antigen retrieval by boiling the sections in an antigen‐unmasking solution. Next, the sections were pre‐treated with 3% H_2_O_2_ in 0.1 m Tris‐buffered saline (TBS, pH 7.4) for 30 min to quench the endogenous peroxidases. The sections were then treated with blocking solution (DAKO, Carpinteria, CA, USA) for 20 min and incubated with anti‐TIPRL or anti‐CD133 antibodies for 30 min in a humidified chamber. After washing with 0.1 m TBST (0.1 m TBS containing 0.1% Tween 20), the sections were incubated with the EnVision anti‐mouse polymer (DAKO) for 30 min. The peroxidases bound to the antibody complex were visualized after treatment with 3,3′‐diaminobenzidine chromogenic substrate solution (DAKO). The 3,3′‐diaminobenzidine reaction was monitored microscopically to determine the optimal incubation time and was stopped with several washes of 0.1 m TBS. The immunolabeled sections were dehydrated in a graded ethanol series, defatted in xylene, and mounted. The sections were examined using an Olympus BX51 microscope (Olympus, Shinjuku‐ku, Tokyo, Japan) under bright‐field illumination, and images were acquired using an Olympus DP70 camera (Olympus).

### Immunofluorescence Microscopy

A549 cells were transfected with hemagglutinin‐tagged CaMKK2‐expressing plasmids [including full‐length N‐terminal (1–165) and C‐terminal (447–540)], siRNAs against TIPRL, or control siRNA. The cells were fixed with 4% paraformaldehyde and analyzed by immunofluorescence staining, as described previously,^[^ ^58^ ^]^ using anti‐TIPRL, anti‐CaMKK2, or anti‐PP2Ac antibodies. The images were captured using a confocal microscope (Carl Zeiss, Oberkochen, Germany).

### Protein Isolation, Immunoblotting, and Immunoprecipitation

Cells were lysed in lysis buffer A (20 mm *N*‐2‐hydroxyethylpiperazine‐*N*′−2‐ethanesulfonic acid [pH 7.5], 150 mm NaCl, 1 mm EDTA, 2 mm ethylene glycol tetra‐acetic acid, 1% Triton X‐100, 10% glycerol, and protease cocktail I/II; Sigma), and cellular debris were removed by means of centrifugation at 10000 × *g* for 10 min. Proteins were separated using sodium dodecyl sulfate‐polyacrylamide gel electrophoresis, transferred onto nitrocellulose membranes, blocked with 5% skim milk in 0.01 m TBS (pH 7.5) containing 0.5% Tween 20, and blotted with the appropriate primary antibodies. Antigen‐antibody complexes were detected using a chemiluminescence analyzer (Western Bright ECL; Advansta, Seoul, South Korea). A549 cells were transfected with an siTIPRL using Lipofectamine RNAiMAX (Thermo Fisher Scientific), according to the manufacturer's protocol, and then subjected to immunoprecipitation. Briefly, cells were rinsed once with cold PBS and then lysed in lysis buffer A. The cell lysates obtained were precleared with 10 µL of protein G sepharose beads (GE Healthcare, Dornstadt, Germany) for 1 h. The cleared lysates were then incubated with 1 µg of antibodies against TIPRL, CaMKK2, or PP2Ac or normal IgG antibodies for 2 h, mixed with 30 µL of Protein G sepharose beads, and then incubated overnight at 4 °C. The beads were washed four times with 1 mL of lysis buffer A, and the precipitated proteins obtained were subjected to immunoblotting.

### qRT‐PCR

Total RNA of human lung cancer tissues, non‐cancerous tissues, and CD133^+^ and CD133^–^ cells was extracted using Trizol reagent (Thermo Fisher Scientific). RNA (1 µg) was reverse‐transcribed using a First‐Strand cDNA Synthesis Kit (Fermentas, Grand Island, NY, USA). All reactions were performed in triplicate, and the *B2M* gene was used as a control. Using the comparative threshold cycle (Ct)^[^ ^59^ ^]^ or standard method, the relative quantification of gene expression was calculated as the ratio of CD133^+^ cells to CD133^–^ cells, after normalizing each sample against B2M. All qRT‐PCR data represent the mean ± SD of two replicates from one of three independent sets of experiments. The primer sequences used in this study are listed in Table S4 (Supporting Information).

### Cut‐And‐Run Assay

Cut‐and‐run experiments were performed according to the manufacturer's protocol (Cut‐and‐run kit; Cell Signaling Technology). Briefly, the cells were harvested, and 250 000 cells per sample were incubated for 10 min with 10 µL of concanavalin A‐coated beads at room temperature. Cells bound to beads were permeabilized and incubated with the appropriate antibody for 1 h, at 4 °C. Protein A‐MNAse at a final concentration of 700 ng mL^−1^ was mixed and incubated with cells for 1 h, at 4 °C. Protein A‐MNAse was activated with 2 µL of 100 mm CaCl_2_, and digestion was performed at 4 °C for 30 min. Release of the fragments was achieved at 37 °C in 15 min, and then the DNA was extracted using Spin columns (Qiagen) and eluted in 30 µL of elution buffer. The extracted DNA was used for qRT‐PCR analysis using SYBR Green Master Mix and CFX96 Touch Real‐Time PCR (Bio‐Rad, Hercules, CA, USA). Data represent the mean ± SD of two replicates from one of three independent experiments. The primers used for qRT‐PCR are listed in Table S4 (Supporting Information).

### Colony‐Formation Assay and Sphere‐Formation Culture

Anchorage‐independent growth was assessed by performing colony‐formation assays in soft agar and sphere‐formation culture. For the colony‐formation assays, cells were suspended in 1 mL of cell growth medium containing 0.3% agar and plated on a layer of 0.6% agar in growth medium. Cells were grown at 37 °C with 5% CO_2_, and the colonies were stained with 0.01% Crystal Violet (Sigma‐Aldrich) for 10 min and counted at 15 days post‐inoculation. In the case of stable cell lines (A549‐sgCont and A549‐sgTIPRL cells), the *EGFP* gene was inserted with sgRNA against the *TIPRL* gene as a selection tool for gene editing using CRISPR/Cas9. The generated colonies and spheroids were analyzed using fluorescence microscopy. For the sphere‐formation culture, the cells were seeded in ultralow‐attachment 6‐well plates, at a density of 10^3^ cells mL^−1^. Sphere cultures were grown in serum‐free DMEM/F‐12 supplemented with 20 ng mL^−1^ epidermal growth factor and 10 ng mL^−1^ basic fibroblast growth factor. Data represent the mean ± SD of three replicates from one of three independent experiments.

### Measurement of Mitochondrial Activity and ATP Production

The specific fluorescent probes MitoSOX, TMRE, and Rhod‐2AM were obtained from Invitrogen. Unsorted and magnetically sorted CD133^+^ and CD133^–^ lung cancer cells were cultured and then incubated with 1 µm MitoSOX for 20 min or with 5 µm TMRE or Rhod2‐AM for 30 min at 37 °C. The signals from the fluorescent probes were quantified using a FACSCanto II flow cytometer (BD Biosciences). Data represent the mean ± SD of two replicates from one of three independent sets of experiments. ATP levels were measured and analyzed using an ATP determination kit (Thermo Fisher Scientific), according to the manufacturer's protocol. Briefly, CD133^+^ and CD133^–^ cells were sorted from lung cancer cell lines and plated at a density of 5 × 10^4^ cells/well in a 12‐well plate. Next, 1 mL of fresh medium (10 mm glucose in fetal bovine serum‐free DMEM/F12) was added for total ATP level measurements. The plates were incubated at 37 °C in a humidified incubator for 90 min. The cells were harvested, lysed, and subjected to an ATP determination assay. ATP levels were quantified using a luminometer (Molecular Devices, Sunnyvale, CA, USA). Data represent the mean ± SD of three replicates from one of three independent experiments.

### Luciferase Assay

Luciferase assays were performed using the Dual‐Luciferase Reporter Assay System (Promega, Madison, WI, USA), according to the manufacturer's specifications. Cells were co‐transfected with TIPRL promoter‐luciferase reporter or CRE minimal promoter plasmids, as well as control Renilla luciferase plasmids, for 24 h and then harvested. The relative luciferase activity was calculated by measuring the luciferase activity and normalizing it to the Renilla luciferase activity. To generate luciferase reporter constructs, the TIPRL promoter (0.5 kb to +20 bp) was PCR‐amplified from normal human genomic DNA (Takara Bio), using primers containing KpnI or HindIII linker sequences, and the PCR products obtained were cloned into the corresponding sites of the pGL4.14 vector (Promega). All reporter assay data represent the mean ± SD of two replicates from one of three independent experiments.

### Evaluation of Tumorigenicity and Toxicity

Tumorigenicity was determined by subcutaneously injecting A549‐sgCont and A549‐sgTIPRL CD133^+^ cells (1 × 10^5^), isolated using magnetic‐activated cell sorting, into the flanks of 6‐week‐old female nude mice. All the institutional and national guidelines for the care and use of laboratory animals were followed (approval no. 2023‐12‐022). All animal procedures were approved by the Institutional Animal Care and Use Committee of the Asan Institute for Life Sciences (Seoul, South Korea). After 10 d, well‐established tumors, with a volume of ≈100 mm^3^ were detected. Four groups (A549‐sgCont, A549‐sgCont+afatinib, A549‐sgTIPRL, and A549‐sgTIPRL+afatinib) were established by pooling five mice from each group. Experimental mice were intraperitoneally injected with afatinib (50 mg kg^−1^ d^−1^, 3 days/week, for 2 weeks), whereas control mice were injected with PBS. The tumor size was measured every 2 days using a digital caliper. Tumor volume (V) was calculated from the measured length (l) and width (w), using the following formula: V = lw^2^/2. The mice were euthanized and sacrificed 28 days after injection, and the tumor samples were analyzed using the DeadEnd Colorimetric TUNEL System (Promega).

For injection of CD133^+^ CSCs into the lungs, the mice were first anesthetized using isoflurane inhalation, and a small nick was made in their skin. The lung was exteriorized, and the lung wall was slightly injected. CD133^+^ cells isolated from A549‐sgCont or A549‐sgTIPRL cells were injected, and the skin was sutured. After 7 weeks, PET‐MRI fusion imaging was performed to assess the tumor burden or number using a NanoPET‐MRI system (1T, Mediso, Budapest, Hungary). The mice were intravenously administered 7.4±1.0 MBq in 0.2 mL of fludeoxyglucose under anesthesia (1.5% isoflurane in 100% O_2_ gas), via the tail vein. MR whole‐body imaging was used to obtain a T1‐weighted with a gradient‐recalled echo 3D sequence (TR = 25 ms, TE_eff_ = 3.4, FOV = 64 mm, matrix = 220 × 220) during the fludeoxyglucose uptake period. Static PET images were acquired over 10 min in a 1–5 coincident in a single field of view with an MRI range. 3D volume of interest (VOI) analysis of the reconstructed images was performed using the InterView Fusion software package (Mediso) and standard uptake value (SUV) analysis. A VOI fixed at a diameter of 2 mm was drawn for the tumor and muscle sites. The SUV of each VOI was calculated using the following formula: SUV_mean_ = [tumor radioactivity in the tumor volume of interest with the unit of Bq/cc × body weight (g)] divided by injected radioactivity.

### Statistical Analysis

Statistical analysis was performed using Student's *t*‐test and SigmaPlot (version 12.0; Systat Software, San Jose, CA, USA). Statistical significance was defined as follows: *P < 0.05, **P < 0.01, and ***P < 0.001. All the data in the study are expressed as mean ± SD of three independent experiments.

## Conflict of Interest

The authors declare no conflict of interest.

## Author Contributions

I.‐S.S., Y.‐J.J., and J.K.Y. contributed equally to this work. I.‐S.S. and S.‐W.J. designed the research. I.‐S.S., Y.J.J., J.K.Y., J.L, H.‐J.Y., Y.‐H.P., S.‐U.K., S.‐M.H., G.D.L, and S.‐W.J. performed the experiments. I.‐S. S., Y.J.J., J.K.Y., H.‐J.Y., Y.‐H.P., S.‐U.K., S.‐M.H., P.C. L., G.D.L., and S.‐W.J. analyzed the data. I.‐S.S. and S.‐W.J. wrote the manuscript.

## Supporting information

Supporting Information

## Data Availability

The data that support the findings of this study are available from the corresponding author upon reasonable request.
