# Supplementary material for: TIPRL Regulates Stemness and Survival in Lung Cancer Stem Cells through CaMKK2‐CaMK4‐CREB Feedback Loop Activation
Source: Adv Sci (Weinh). 2024 Jul 30;11(36):2406309. doi: 10.1002/advs.202406309 (PMC11423089; doi:10.1002/advs.202406309)
Supplement: Supplementary file 1 — Supporting Information [file ADVS-11-2406309-s001.docx]

Supporting Information

TIPRL regulates stemness and survival in lung cancer stem cells through CaMKK2-CaMK4-CREB feedback loop activation

In-Sung Song, Yu-Jeong Jeong, Jae Kwang Yun, Jimin Lee, Hae-Jun Yang, Young-Ho Park, Sun-Uk Kim, Seung-Mo Hong, Peter C.W. Lee, Geun Dong Lee*, and Sung-Wuk Jang*


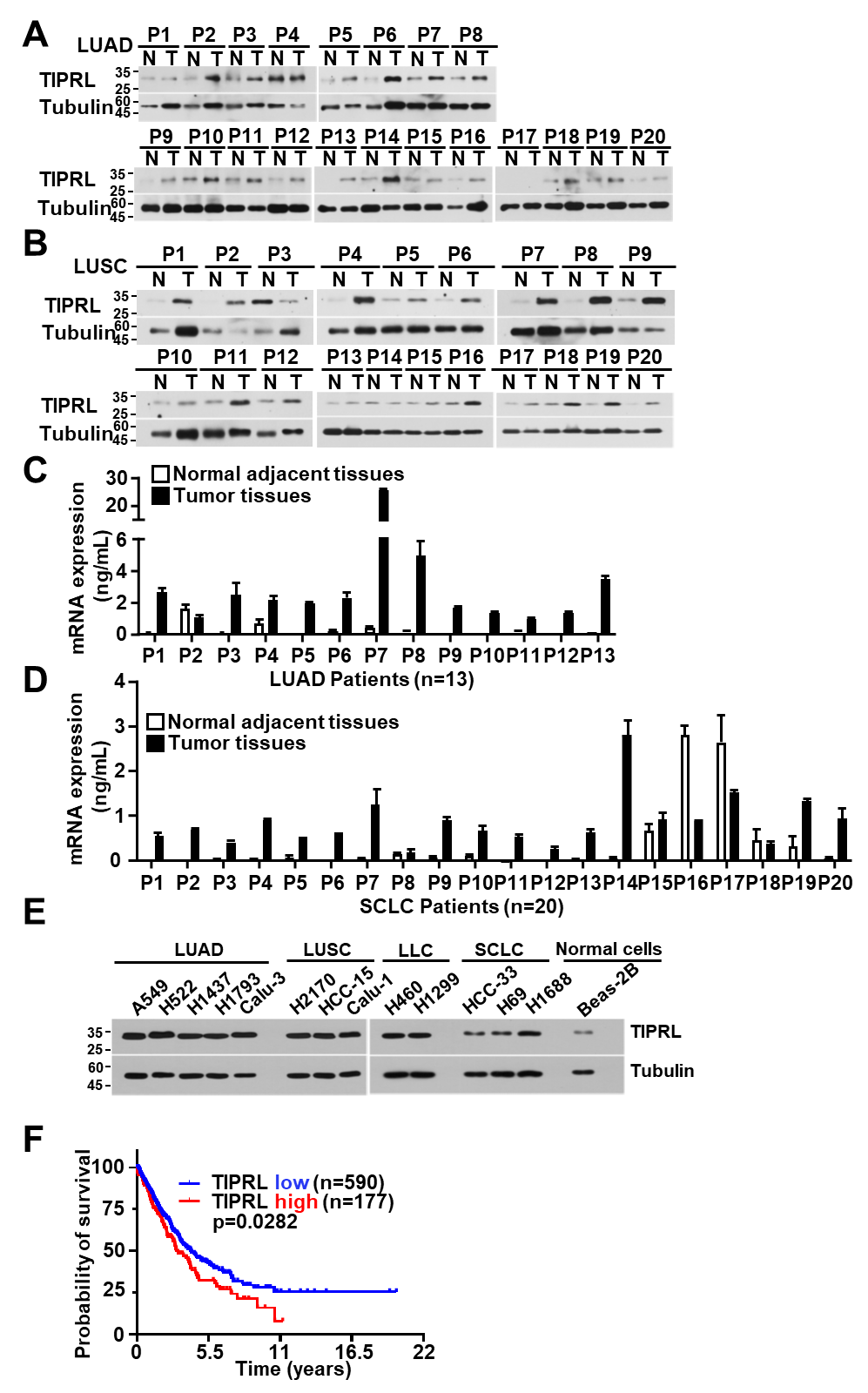


Figure S1 TIPRL is upregulated in tissues from patients with LUAD, LUSC, and SCLC. (A, B) Immunoblotting with an antibody against TIPRL performed in 20 pairs of tissues from patients with LUAD (A) and LUSC (B). T represents a tumor tissue, while N represents a normal tissue in the lung. (C, D) Quantitative real-time PCR analysis of the transcriptional level of TIPRL in cancerous and adjacent normal tissues from patients with LUAD (*n* = 13) and SCLC (*n* = 20). (E) TIPRL expression levels determined using immunoblotting in lung cancer cell lines. (F) Kaplan–Meier plot comparing disease-free survival in patients with high *versus* low expression levels of TIPRL in TCGA lung cancer cohort.

LUAD, lung adenocarcinoma; SCLC, small cell lung cancer; LUSC, lung squamous cell carcinoma; TIPRL, target of rapamycin signaling pathway regulator; TCGA, The Cancer Genome Atlas


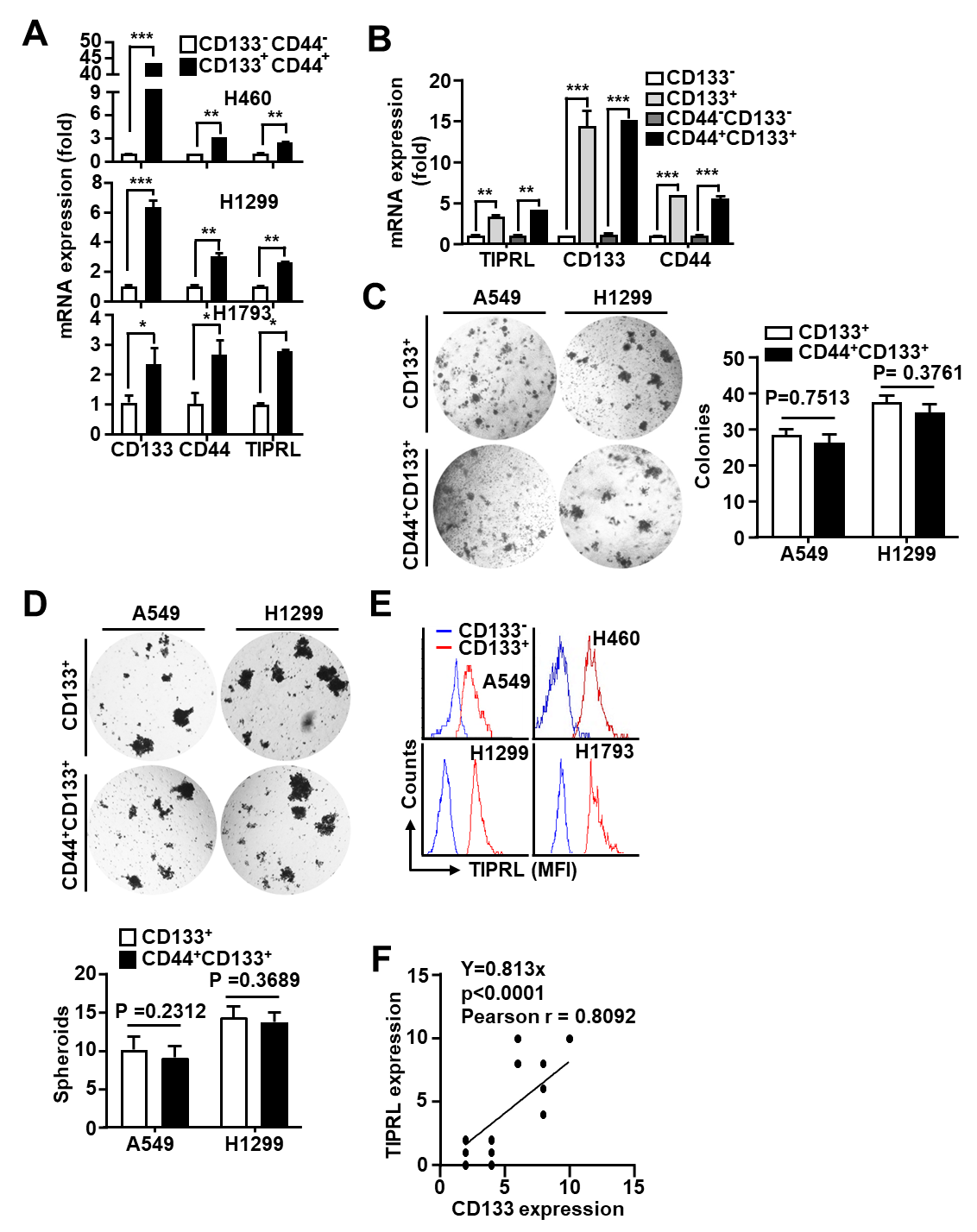


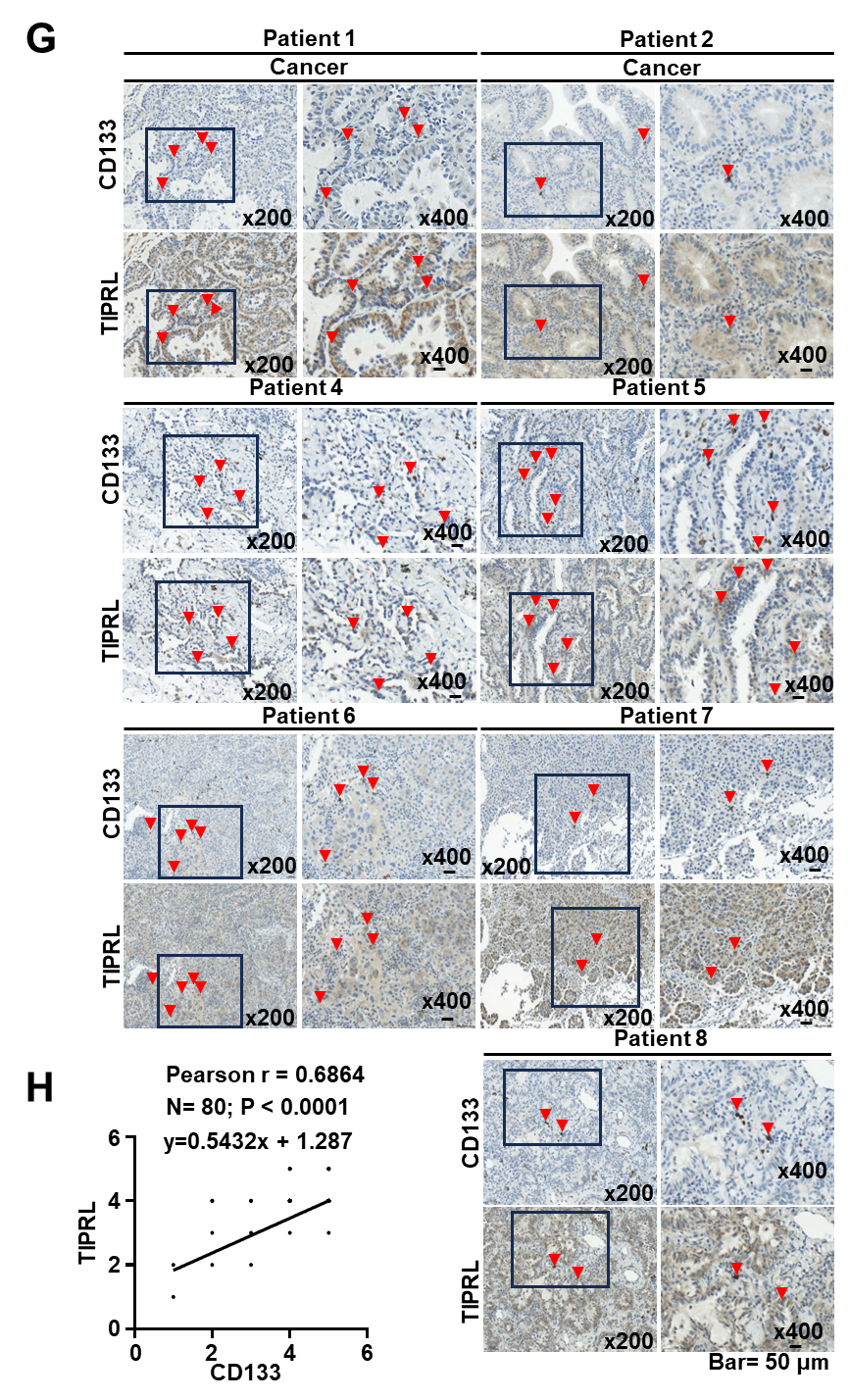


Figure S2 TIPRL is upregulated in CSCs isolated from tissues of patients with lung cancer. (A) mRNA expression levels in lung cancer cells divided into CSCs and non-CSCs, measured using quantitative real-time PCR. (B–D) Comparison of mRNA expression levels (B), colony-forming ability (C), and sphere-forming ability (D) between CD133^+^ and CD44^+^CD133^+^ subpopulations isolated from A549 cells using either CD133 antibody or CD44/CD133 antibodies, respectively. (E) Lung cancer cells were stained with antibodies against TIPRL and CD133, and TIPRL expression was analyzed in CD133^−^ and CD133^+^ population using FACScantoII. (F) Analysis of the correlation between TIPRL and CD133 mRNA expression, as measured in Figure 1F, using tissues from 8 patients with lung cancer. (G, H) Expression of TIPRL and CD133 in lung cancer tissues from 7 patients, determined using immunohistochemistry (G); red arrows indicate highly positive cells. (H) Analysis of the correlation between TIPRL and CD133 expression using the immunohistochemistry data.

TIPRL, target of rapamycin signaling pathway regulator; CD, cluster of differentiation; CSCs, cancer stem cells


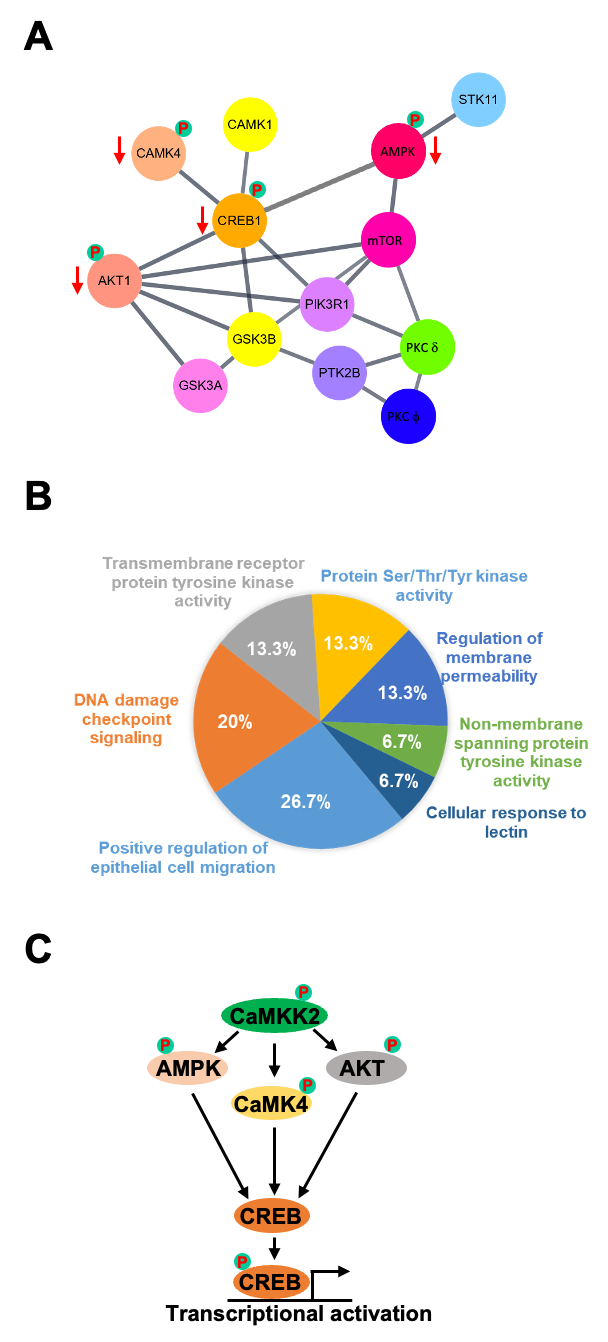


Figure S3 TIPRL depletion reduces the activity of diverse kinase in lung cancer cells. (A–C) Phospho Explorer Antibody Array and bioinformatic analysis of TIPRL-depleted A549 cells. (A) STRING network of known protein-protein interactions among the 13 proteins that showed altered phosphorylation. (B) Functional classification of proteins with altered phosphorylation induced by TIPRL depletion, determined using the ClueGO system (C) Schematic illustration of the mechanism through which TIPRL mediates the function of CaMKK2 in lung cancer cells.

TIPRL, target of rapamycin signaling pathway regulator; siRNA, short interfering RNA


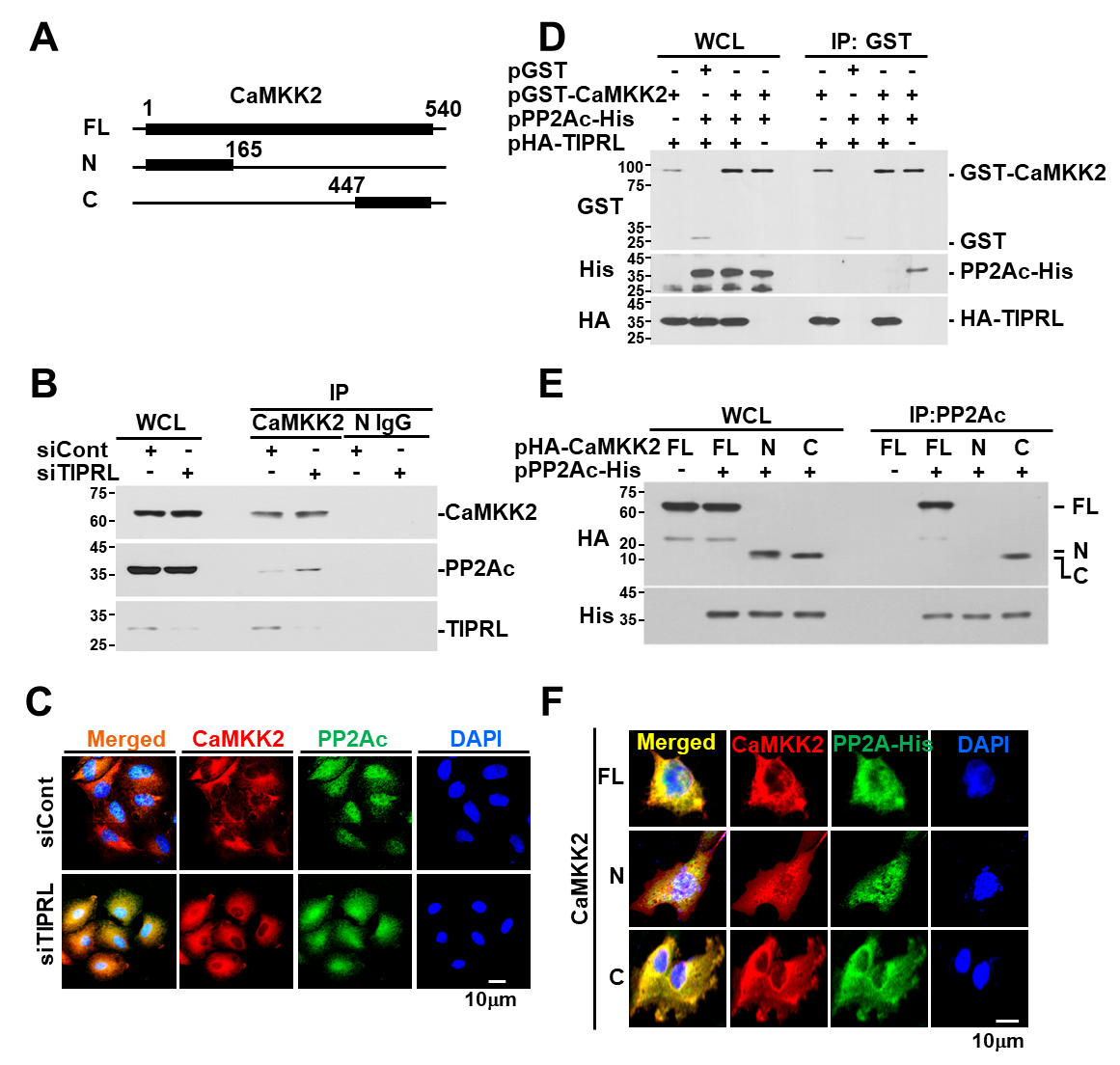


Figure S4 CaMKK2 activation is maintained by blocking its interaction with the protein phosphatase, PP2Ac, facilitated by TIPRL. (A) Full-length as well as C-terminal-deleted (N-form) and N-terminal-deleted (C-form) forms of the CaMKK2-expressing plasmid. (B) IP assay and ICC (C) results for A549 cells transfected with an siRNA against TIPRL or control siRNA. (D) IP assay of HEK293 cells co-transfected with pHA-CaMKK2, pTIPRL-His, and pGST-PP2Ac using a GSH bead. (E) IP assay of HEK293 cells co-transfected with pPP2Ac-His and pHA-CaMKK2 or deletion mutants using an antibody against PP2Ac protein. (F) Confocal microscopic images of HEK 293 cells co-transfected with pPP2Ac-His and pHA-CaMKK2 or deletion mutants and co-stained with antibodies against hemagglutinin and His tags.


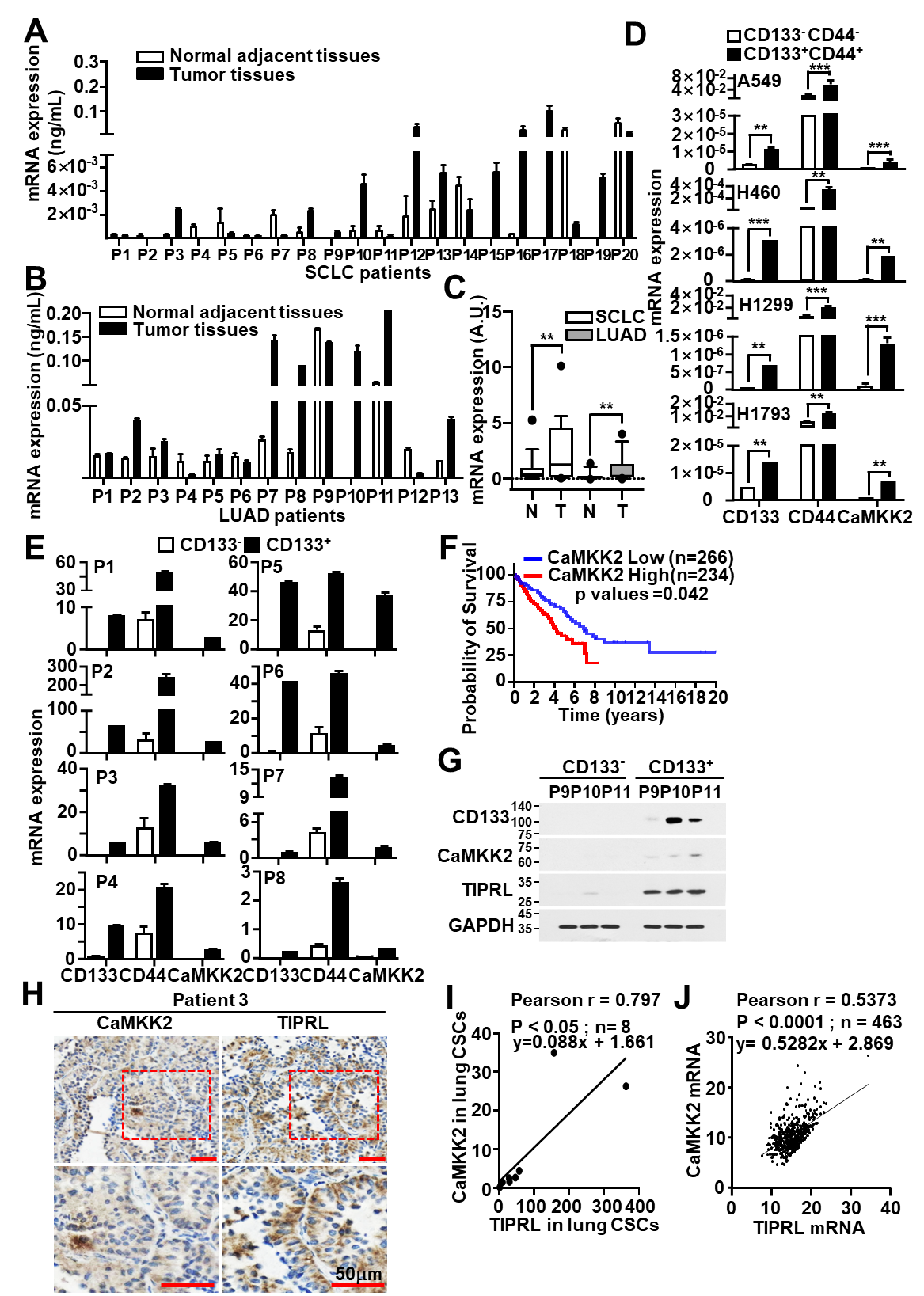


Figure S5 CaMKK2 is upregulated in CSCs isolated from tissues of patients with lung cancer and is associated with poor prognosis. Quantitative real-time PCR analysis of the mRNA expression of CaMKK2 in tissues of patients with (A) SCLC and (B) LUAD; (C) box plot showing the median, 25^th^, and 75^th^ percentiles. Quantitative real-time PCR analysis of the mRNA level of CaMKK2 in lung cancer cell lines (D) and freshly acquired tissues from patients with lung cancer (*n* = 8) sorted into CSCs and non-CSCs (E); values were normalized to the expression of β2-microglobulin. (F) Kaplan–Meier plot comparing the disease-free survival in patients with high *versus* low expression levels of CaMKK2, using TCGA lung cancer cohort.

(G) Immunoblotting to determine CaMKK2 and TIPRL expression in lung CSCs freshly isolated from patients with lung cancer. (H) CaMKK2 and TIPRL expression in lung cancer tissues from patient 3, determined using immunohistochemistry. Correlation between TIPRL and CaMKK2 expression in lung CSCs acquired from patients with lung cancer (I, *n* = 8) and in TCGA lung cancer cohort (J).

TIPRL, target of rapamycin signaling pathway regulator; TCGA, The Cancer Genome Atlas; CD, cluster of differentiation; CSCs, cancer stem cells; LUAD, lung adenocarcinoma; SCLC, small cell lung cancer; CaMKK2, calcium/calmodulin-dependent protein kinase kinase 2


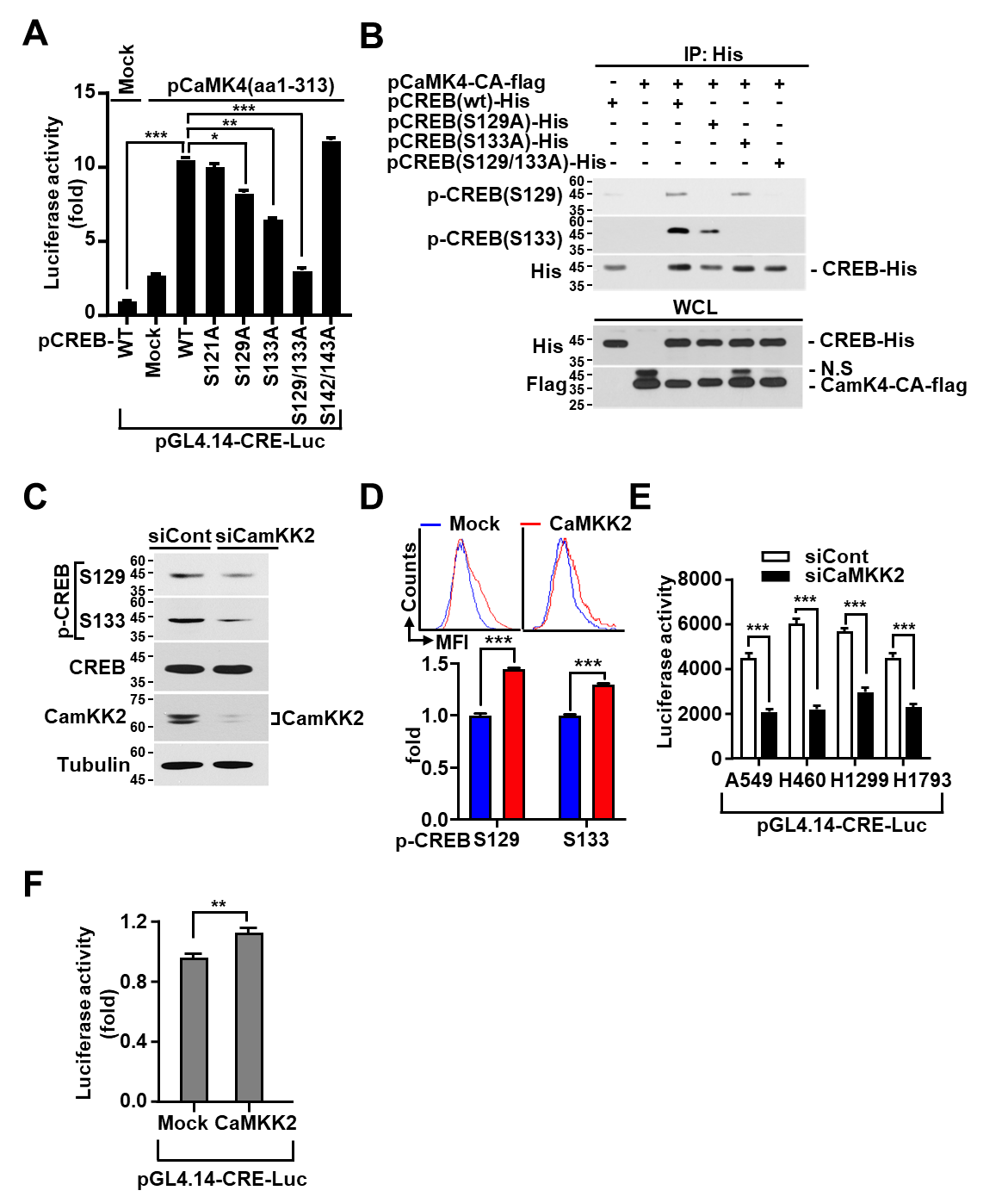


Figure S6 CaMKK2 phosphorylates CaMK4, which phosphorylates CREB at Ser129 and Ser133, thus activating CREB target genes. (A) Dual-Luciferase^®^ assay of HEK 293 cells co-transfected with a CRE-driven luciferase reporter and pCaMK4/CREB mutants. (B) Immunoblotting to determine CREB phosphorylation in HEK 293 cells co-transfected with pCaMK4-flag and wild-type or mutant forms of CREB-His (CREB S129A, S133A, and S129/133A). (C) Immunoblotting of A549 cells transfected with an siRNA against CaMKK2 and control siRNA. (D) FACSCanto™ II analysis of A549 cells transfected with pHA-CaMKK2 and mock, using phospho-CREB antibodies. (E) Dual-Luciferase^®^ assay of A549, H460, H1299, and H1793 lung cancer cells co-transfected with cAMP response element-driven luciferase reporter plasmids and siControl or siCaMKK2. (F) Luciferase assay of A549 cells transfected with pHA-CaMKK2 and cAMP response element-driven luciferase reporter plasmids.

CaMKK2, calcium/calmodulin-dependent protein kinase kinase 2; CaMK4, calcium/calmodulin-dependent protein kinase 4; CREB, cAMP response element-binding protein

Figure S7 TIPRL depletion suppresses the tumorigenic ability in lung cancer. (A) Migration and (B) wound-healing assays of siTIPRL-transfected A549 and H1299 cells. (C) FACSCanto™ II flow cytometric analysis of A549 cells transfected with an siRNA against TIPRL or control siRNA and infected with viral particles containing TIPRL or mock for identifying the CD133^+^ population. (D) Invasion and (E) wound-healing assays of A549 and H1299 cells transfected with an siRNA against TIPRL gene and control siRNA and infected with lentiviral particles against TIPRL or Mock. Invasion data represent the means ± SD of triplicate. Wound-healing assay data represent the mean ± SD of results taken at five points in one of three independent experiments. (F) Measurement of ATP levels in A549, H1299, H460, and H1793 lung cancer cells transfected with an siRNA against TIPRL or control siRNA.

TIPRL, target of rapamycin signaling pathway regulator; siRNA, short interfering RNA


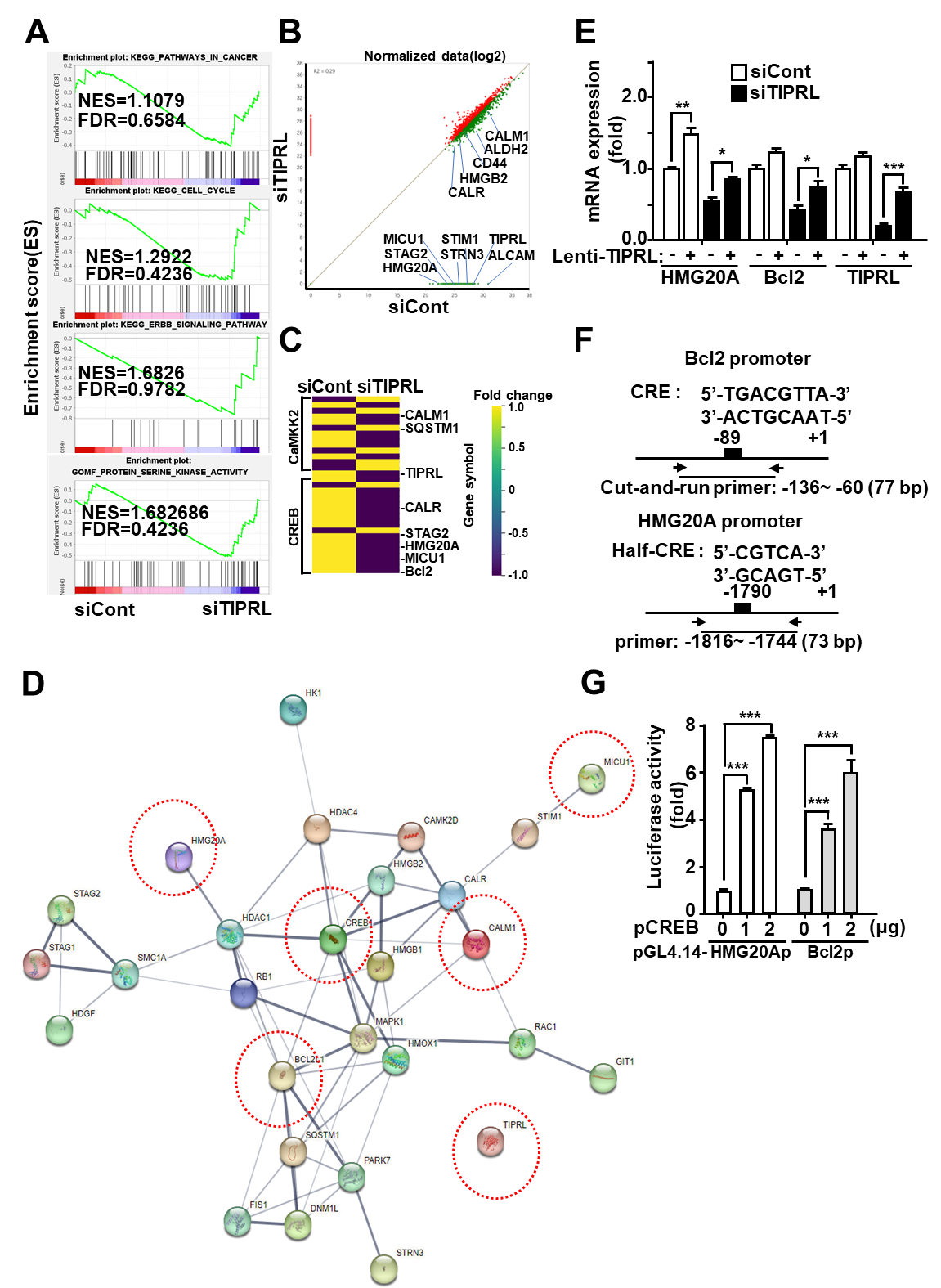


Figure S8 CaMKK2-CaMK4-CREB signaling axis regulates the expression of the CREB target genes Bcl2 and HMG20A in lung cancer. (A–D) Liquid chromatography with tandem mass spectrometry and bioinformatic analysis of proteins showing altered expression following TIPRL depletion. (A) Four most common functional gene sets enriched in the TIPRL-depleted samples in GSEA conducted to search for Kyoto Encyclopedia of Genes and Genomes; enriched gene sets were “cancer”, “cell cycle”, “ERBB signaling pathway”, and “serine kinase activity”. (B) Illustration of the scatter plot; values of the x-coordinate and y-coordinate in the scatter plot are normalized signal values. (C) Hierarchical clustering generated by the CaMKK2 signaling pathway-related proteins. (D) STRING network showing the association of proteins regulated upon TIPRL depletion; red-dot circles represent the key proteins downregulated upon TIPRL depletion. (E) qRT-PCR analysis of mRNA expression in A549 cells transfected with siTIPRL and siCont and then infected with TIPRL-expressing viral particles. (F) Illustration of the qPCR primer location and CRE-binding site on the promoter of each gene used in the cut-and-run assay. (G) Luciferase reporter assay of A549 cells co-transfected with HMG20A and Bcl2-promoter-driven reporter plasmids as well as pHA-CREB plasmids.

TIPRL, target of rapamycin signaling pathway regulator; CRE, cAMP response element; GSEA, Gene Set Enrichment Analysis


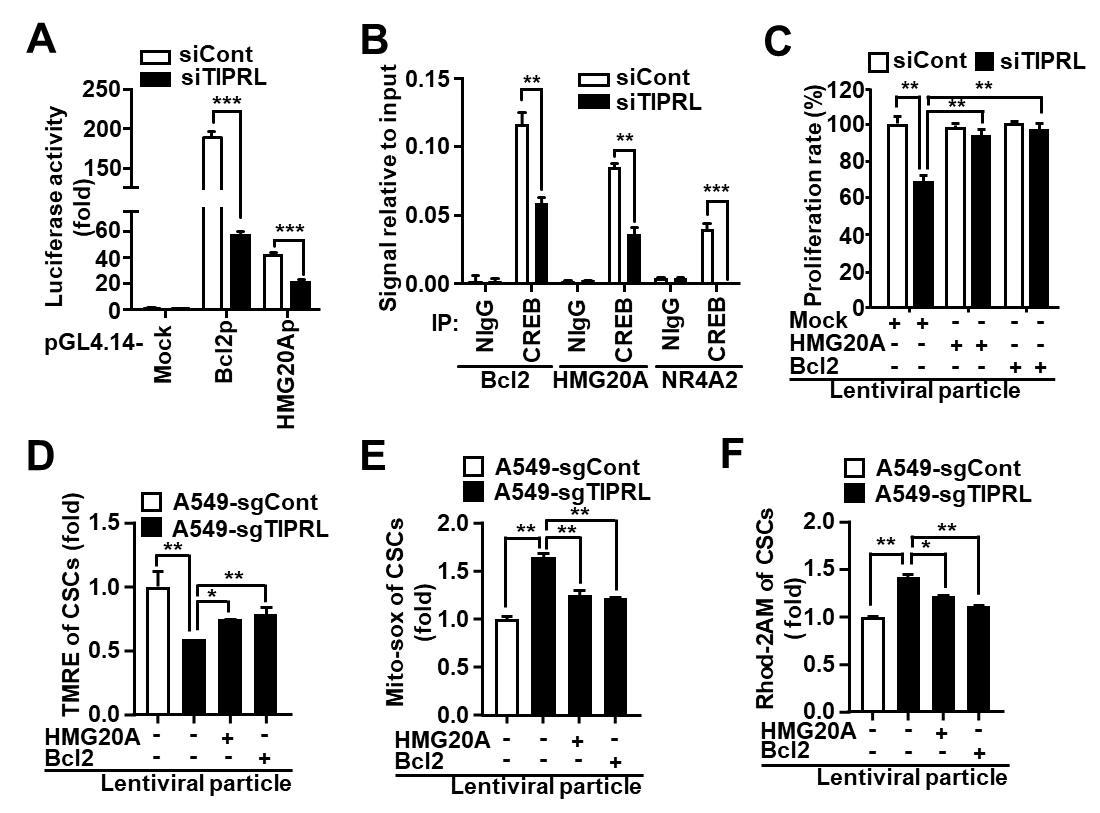


Figure S9 Relationship of Bcl2 and HMG20A expression with TIPRL depletion and mitochondrial function

(A) Luciferase activity in siTIPRL-transfected A549 cells co-transfected with *Bcl2* or *HMG20A* promoter-driven-luciferase reporter plasmids. (B) qRT-PCR analysis of the association of CREB with the *Bcl2* and *HMG20A* promoters in TIPRL-depleted A549 cells following cut-and-run assay using an antibody against CREB; *NR4A2* was used as a positive control for the CREB target genes. (C) CCK-8 assay to identify the CD133^+^ population of CSCs in TIPRL-depleted A549 cells infected with *HMG20A*, *Bcl2*, or mock lentivirus. Measurement of (D) the mitochondrial membrane potential (TMRE staining), (E) ROS levels (MitoSOX staining), and (F) Ca^2+^ levels (Rhod2-AM staining) in CD133^+^ and CD133^–^ A549-sgTIPRL and control cell lines infected with lentiviral particles against *HMG20A* and *Bcl2* genes for 24 h.

TIPRL, target of rapamycin signaling pathway regulator; CCK-8, Cell Counting Kit-8; CREB, cAMP response element-binding protein; CD, cluster of differentiation; CSCs, cancer stem cells; TMRE, tetramethylrhodamine


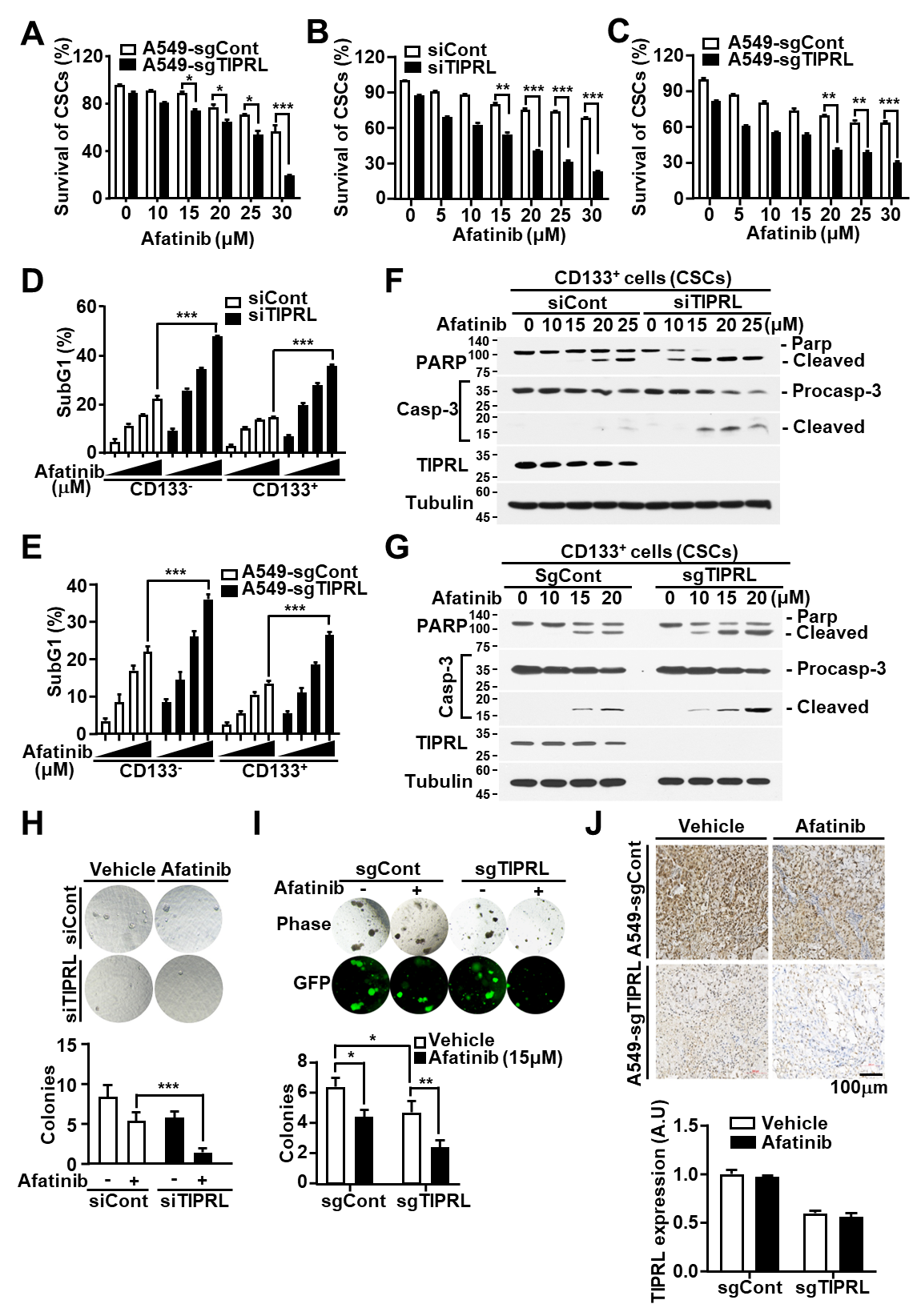


Figure S10 TIPRL depletion enhances afatinib-induced cell death in lung CSCs. (A) FACSCantoII™ analysis of death of lung CSCs using CD133^–^ and CD133^+^ A549-sgCont/sgTIPRL cells treated with afatinib and stained with the Annexin V-FITC/PI reagent. Cell Counting Kit-8 assay for measuring cytotoxicity in (B) siTIPRL-transfected A549 cells and (C) the A549-sgTIPRL cell line. Determination of early apoptotic cell death in terms of the subG1 hypodiploid phase by analysis of DNA content in (D) siTIPRL-transfected A549 cells and (E) the A549-sgTIPRL cell line. Immunoblotting analysis of caspase-3 and poly (ADP-ribose) polymerase cleavage levels in (F) transiently TIPRL-depleted A549 cells and (G) the A549-sgTIPRL cell line. Soft agar assay of (H) siTIPRL-transfected A549 cells and (I) the A549-sgTIPRL cell line treated with 20 μM afatinib for 24h and then incubated for 3 weeks with 20 ng mL^−1^ epidermal growth factor and 10 ng mL^−1^ basic fibroblast growth factor; colony and spheroid counts are shown using a fluorescence microscope. (J) Expression of TIPRL in xenograft tumors generated with A549-sgCont and A549-sgTIPRL cells (each group, n = 5) in Figure 6D was determined using immunohistochemistry. The immunostaining data were quantified and represented in the graph.

CSCs, cancer stem cells; TIPRL, target of rapamycin signaling pathway regulator; CD, cluster of differentiation; FITC, fluorescein isothiocyanate; PI, propidium iodide

Table S1. Genetic alteration and histological type of Lung Cancer and Benign Cell Lines Used in the Study

| **Cell Line** | **Cancer Type** | **KRAS** | **TP53** | **EGFR** | **Other Mutations** |
| --- | --- | --- | --- | --- | --- |
| **A549** | LUAD | G12S mutation | Mutation | Wild-type | - |
| **H23** | LUAD | G12C mutation | R248L mutation | Wild-type | - |
| **H1793** | LUAD | Wild-type | R273L mutation | Amplification/mutations in some sublines | - |
| **H2170** | LUSC | - | Various mutations | - | PIK3CA mutation, CDKN2A deletion/mutation |
| **Calu-1** | LUSC | Wild-type | Homozygous deletion | - | RB1 mutation/deletion |
| **H1299** | Large Cell Carcinoma | Wild-type | Null (complete deletion) | - | NRAS mutations |
| **H460** | Large Cell Carcinoma | Q61H mutation | Wild-type | - | RB1 mutation/deletion |
| **H1688** | SCLC | - | Mutation | - | MYC amplification, RB1 inactivation |
| **HEL299** | Normal Epithelial | - | - | - | Normal human embryonic lung tissue |
| **LL24** | Normal Epithelial | - | - | - | Normal human lung fibroblasts |
| **Beas-2B** | Normal Epithelial | - | - | - | Immortalized with adenovirus 12-SV40 hybrid virus |

**Note**: "-" indicates that no mutations or changes were reported for the gene, or that no information is available for that specific gene in the cell line

Table S2. Antibodies used in the current study

| Target | Antibody type | Source | Identifier | Dilution in | | |
| --- | --- | --- | --- | --- | --- | --- |
|  |  |  |  | WB | IHC/  ICC | Cell sorting |
| TIPRL | Rabbit polyclonal | Bethyl | A300-663A | 1:2000 | 1:150 |  |
| CaMKK2 | Rabbit polyclonal | CST* | #16810 | 1:1000 | 1:150 |  |
| PP2Ac | Mouse monoclonal | Santa Cruz | SC-80665 | 1:1000 | 1:150 |  |
| Phosphor-Ser | Mouse monoclonal | Invitrogen | MA190649 | 1:1000 |  |  |
| CaMK4 | Mouse monoclonal | Santa Cruz | SC-55501 | 1:1000 |  |  |
| CREB1 | Rabbit monoclonal | CST | #9197 | 1:1000 |  |  |
| p-CREB  (S129) | Rabbit polyclonal | Invitrogen | PA5-36843 | 1:1000 |  |  |
| p-CREB  (S133) | Mouse monoclonal | Santa Cruz | SC-81486 | 1:1000 |  |  |
| CD133 | Mouse monoclonal | Miltenyi | 130-092-395 | 1:100 | 1:100 |  |
| CD133-APC | Mouse monoclonal | Miltenyi | 130-113-106 |  |  | 1:50 |
| CD133-microbead | Mouse monoclonal | Miltenyi | 130-097-049 |  |  | 10 μL/10^7^ |
| CD44-PE | Mouse monoclonal | Miltenyi | 130-113-342 |  |  | 1:50 |
| HA | Rat polyclonal | Roche | 11867423001 | 1:1000 |  |  |
| His | Mouse monoclonal | Qiagen | 34660 | 1:2000 | 1:100 |  |
| GST | Mouse monoclonal | Santa Cruz | SC-138 | 1:3000 |  |  |
| Flag | Mouse monoclonal | Sigma- Aldrich | F3165 | 1:3000 |  |  |
| Bcl2 | Mouse monoclonal | Santa Cruz | SC-7382 | 1:500 |  |  |
| HMG20A | Mouse monoclonal | Santa Cruz | SC-393028 | 1:1000 |  |  |
| Cleaved Caspase-3 | Rabbit monoclonal | CST | #9664 | 1:1000 |  |  |
| Caspase-3 | Mouse monoclonal | Santa Cruz | SC-56053 | 1:1000 |  |  |
| PARP | Rabbit polyclonal | CST | #9542 | 1:2000 |  |  |
| Tubulin | Mouse monoclonal | Santa Cruz | SC-23948 | 1:3000 |  |  |
| Histon H3 | Mouse monoclonal | Santa Cruz | SC-517576 | 1:1000 |  |  |
| Mouse-IgG, HRP | Goat polyclonal | Invitrogen | 31430 | 1:3000 |  |  |
| Rabbit-IgG, HRP | Goat polyclonal | Invitrogen | 31463 | 1:3000 |  |  |
| Rat-IgG, HRP | Goat polyclonal | Invitrogen | 31470 | 1:3000 |  |  |

*CST, Cell Signaling Technology

Table S3. Patient descriptions and tumor features

| Patient | Age/Sex | Site | Clinical stage | EGFR | Histology | TNM | Tumor size (mm) | CD133 % |
| --- | --- | --- | --- | --- | --- | --- | --- | --- |
| P1 | 52/F | RML* | Ιa2 | mutation | Invasive AD* | T1bN0M0 | 15 × 13 × 12 | 1.5 |
| P2 | 63/F | RML* | Ιa2 | mutation | Invasive AD* | T2aN0M0 | 48 × 44 × 32 | 0.8 |
| P3 | 83/M | RUL* | Ιa3 | mutation | Invasive AD* | T2aN0M0 | 27 × 18 × 6 | 0.6 |
| P4 | 67/F | RUL* | Ιa2 | unknown | Invasive AD* | T1cN0M0 | 22 × 20 × 18 | 1.0 |
| P5 | 68/F | LUL* | Ιb | mutation | Invasive AD* | T2aN0M0 | 40 × 16 × 14 | 1.1 |
| P6 | 43/F | LUL* | Ιa3 | unknown | Invasive AD* | T2aN0M0 | 33 × 15 × 10 | 0.9 |
| P7 | 64/M | LUL* | Ιa2 | None | Invasive AD* | T1bN0M0 | 16 × 15 × 10 | 1.2 |
| P8 | 68/F | LUL* | Ιb | None | Invasive AD* | T2aN0M0 | 29x26 × 22 | 1.4 |
| P9 | 69/M | LUL | IIb | mutation | Invasive AD* | T1cN1M0 | 27×24×21 | 2.1 |
| P10 | 64/M | RLL | IIb | None | Invasive AD* | T2aN2M0 | 38×36×35 | 1.8 |
| P11 | 67/M | LLL | IIa | unknown | Invasive AD* | T1bN0M0 | 19×17×9 | 1.6 |

*AD, adenocarcinoma; RML, right middle lobe; RUL, right upper lobe; LUL, left upper lobe

Table S4. qPCR primer sequences used in the current study

| Gene | Accession  number | Primer sequences | | Product size (bp) |
| --- | --- | --- | --- | --- |
|  |  | Forward | Reverse |  |
| TIPRL | NM_152902.5 | tgatgatccacggcttccag | ccctccgtcctgctttcttg | 292 |
| CaMKK2 | NM_006549.4 | cgctggtcgaagtgactgaag | ggttgtcttcgctgccttgc | 235 |
| CD133 | NM_006017 | gcaatctccctgttggtgat | tcagatctgtgaacgccttg | 217 |
| CD44 | NM_000610.3 | tctgtgcagcaaacaacaca | tagggttgctggggtagatg | 234 |
| CREB1 | NM_004379.5 | cagggcctgcaaacattaacc | acaactccaggggcaatagtg | 197 |
| Bcl2 | NM_000633 | atgtgtgtggagagcgtcaa | acttcacttgtggcccagat | 255 |
| HMG20A | NM_018200.4 | cgctaccttgatgaagcagac | cgagctttgctatggttcaag | 245 |
| GAPDH | NM_002046.7 | ctctgctcctcctgttcgac | aatccgttgactccgacctt | 105 |
| B2M | NM_004048.2 | ctcgctccgtggccttag | caaatgcggcatcttcaa | 390 |
| qPCR primer sequences used in the cut-and-run assay | | | | |
| NR4A2 | NM_006186 | atagcgcgggctgcgc | gactcggccccgcggcca | 79 |
| TIPRL | NM_152902.5 | ccttccctggcagtccctaaa | aaggtgtggttaggaacgcacc | 82 |
| Bcl2 | NM_000633 | ggctcagaggagggctct | agcccgaccggtttcctg | 77 |
| HMG20A | NM_018200.4 | ggcccatttgggcgtc | catgactacatattagcgtcctcg | 83 |
